# Implementing childhood cancer survivorship care plans: qualitative perspectives and experiences from Australian primary care physicians

**DOI:** 10.1136/fmch-2026-004002

**Published:** 2026-11-05

**Authors:** Joseph Elias, Jordana K McLoone, Christina Signorelli, Shuang Liang, Skye McKay, Joel Rhee, Elysia Thornton-Benko, Renae Lawrence, Claire E Wakefield, Richard J Cohn, Carolyn Mazariego, Natalie Taylor

**Affiliations:** 1 Implementation to Impact, School of Population Health, University of New South Wales Medicine and Health, Sydney, New South Wales, Australia; 2 The Minderoo Children’s Comprehensive Cancer Centre, Sydney Children’s Hospital Randwick, Randwick, New South Wales, Australia; 3 Behavioural Sciences Unit, Discipline of Paediatrics and Child Health, School of Clinical Medicine, University of New South Wales Medicine and Health, Sydney, New South Wales, Australia; 4 Discipline of General Practice, School of Clinical Medicine, University of New South Wales Medicine and Health, Sydney, New South Wales, Australia; 5 Discipline of Paediatrics and Child Health, School of Clinical Medicine, University of New South Wales Medicine and Health, Sydney, New South Wales, Australia; 6 Bondi Road Doctors, Sydney, New South Wales, Australia; 7 Division of Quality of Life and Pediatric Palliative Care, Stanford University Department of Pediatrics, Stanford, California, USA

**Keywords:** Health Plan Implementation, Medical Oncology, Physicians, Primary Care, Qualitative Research, Pediatrics

## Abstract

**Objective:**

To explore primary care physicians’ (PCPs) perspectives and experiences with implementing cancer survivorship care plans developed through ‘Engage’—a multi-disciplinary intervention for survivors of childhood brain cancer.

**Design:**

Qualitative interview study, guided by implementation science frameworks and a ‘think aloud’ exercise using a real-world survivorship care plan, conducted during a hybrid effectiveness-implementation trial of ‘Engage’.

**Participants:**

20 Australian PCPs participated in interviews, 3 of whom had direct experience referring a patient to participate in ‘Engage’, with the remainder recruited from the general workforce and invited to review the same sample survivorship care plan, based on a real childhood brain cancer survivor from ‘Engage’.

**Setting:**

Most PCPs practised in a private (90%) metropolitan (75%) setting, with representation from seven of eight Australian states and territories.

**Analysis:**

Deductive coding was guided by the constructs of the Consolidated Framework for Implementation Research V.2.0 (CFIR) and the Theoretical Domains Framework. An inductive approach was applied to develop themes reflecting participants lived experiences. Conventional content analysis was also undertaken to quantify participants’ responses regarding preferred formats, topics and dissemination pathways for educational materials for PCPs.

**Results:**

PCPs’ suggestions to improve the content and structure of the ‘Engage’ care plan (3/20 ‘Engage’ PCPs and 17/20 general workforce PCPs) included offering a clearer rationale for follow-up recommendations, providing specialists contact information and using tables and visual cues to improve information salience (CFIR construct: Innovation Design). Key barriers to implementing the care recommended by the plan included lack of access to community care and medical specialists (Local Conditions), practice software constraints (Information-Technology Infrastructure), time pressures (Opportunity) and difficulties engaging survivors (Opportunity, Motivation). Facilitators included support from a specialist nurse at the ‘Engage’ survivorship clinic for referrals and education (Partnerships and Connections) and strategies to improve patient engagement in care (Engaging – Innovation Recipients). Most PCPs (18/20) expressed a desire for tailored survivorship care educational resources (Access to Knowledge and Information). While attitudes toward shared care approaches reported by a small subset of PCPs were positive (5/20, Implementation Process), operationalisation challenges were anticipated with its implementation.

**Conclusions:**

We underscore the critical role of PCPs in the care trajectory of paediatric survivors and address a major gap in their inclusion in survivorship research and practice. Our findings offer practical recommendations for improving survivorship care plan design, alongside the broader system, practice and individual-level changes required for effective implementation.

**Trial registration number:**

The Australian and New Zealand Clinical Trials Registry (ANZCTR), https://www.anzctr.org.au, (ACTRN12621000590864).

**WHAT IS ALREADY KNOWN ON THIS TOPIC**

- Treatment advances have led to a growing paediatric cancer survivorship cohort, prompting a shift from increasingly unsustainable specialist-led care to primary care management of low-risk survivors.
- Survivorship care plans provide primary care physicians with a summary of a survivors’ cancer diagnosis and treatment, risk of late effects and recommended follow-up care.

**WHAT THIS STUDY ADDS**

- This study uncovers factors impacting the implementation of survivorship care plans in primary care.
- Our findings identify physicians’ design preferences, barriers and facilitators to care plan use across system, practice and individual levels, as well as capturing physicians’ attitudes toward shared care approaches.

**HOW THIS STUDY MIGHT AFFECT RESEARCH, PRACTICE OR POLICY**

- This study advances survivorship care research by applying implementation science frameworks to specify key theoretical constructs influencing care plan use and implementation, thereby providing a common language to guide and propagate behaviour change.
- This study proposes adaptations to care plan design and offers practical implementation recommendations at provider, patient, practice and systems levels, with implications for policymakers to support broader structural change.

## Introduction

As global cancer incidence and likelihood for survival increases, so too does the survivorship population, and more survivors than ever before are facing life-impacting chronic health conditions related to their cancer and its treatment.^1–3^ Treatment-related sequelae may not become apparent until long after treatment completion, sometimes emerging years or decades later.^2^ A population that requires particular attention are paediatric cancer survivors who, due to their risk of developing late-effects in early adulthood, require holistic, person-centred, risk-based and evidence-informed follow-up care across primary, community-based and specialist healthcare levels.^4^

Since landmark recommendations were made by the Institute of Medicine in 2006, a critical component of follow-up care has involved the provision of a Survivorship Care Plan (SCP).^5^ An SCP is a personalised summary of care needs developed by an oncology provider once their patient ceases active treatment and enters extended survival. The summary should contain information about the survivor’s cancer diagnosis, cancer treatment received, potential late effects, health behaviour advice, recommended action points for current care and a plan for follow-up/surveillance.^5^ SCPs are also used to document how care is coordinated for survivors who require involvement from a range of health systems and services.

Primary care physicians (PCPs) believe they should play a critical role in ongoing survivorship care.^6^ Yet PCPs report feeling unprepared to manage the long-term care of paediatric survivors, underscoring the need for additional supports.^6^ Many PCPs view care plans as tools that can enhance their knowledge and improve communication with tertiary care providers, providing additional confidence.^6 7^ Survivors and their families also find care plans helpful and educational;^8^ however, many are reluctant for PCPs to assume a central role and often disengage from their PCP during cancer treatment and the survivorship period.^9 10^ In practice, PCPs are eventually or concurrently involved to coordinate aspects of cancer late effects care, particularly when survivors age out of paediatric care and transition to the adult healthcare system.^11^ Notable advantages of including PCPs within the survivors’ care team include holistic cancer and non-cancer related continuity of care, greater accessibility and ability to provide care within a survivors’ own community.^12 13^ However, in Australia and New Zealand few paediatric survivors (~22%) and PCPs (~12%) recall receiving SCPs.^13 14^ Among those who do, evaluations of patients’ reported outcomes (eg, satisfaction, distress, self-efficacy, quality of life) have yielded mixed results.^15–18^ Understanding the challenges physicians face in delivering survivorship care, and their preferences for SCP design, may improve their implementation and inform strategies to build clinician capacity and confidence. Such supports may strengthen survivors’ self-efficacy and, over time, increase trust in their PCPs.

The USA’s National Cancer Institute’s Division of Cancer Control supports the use of implementation science to advance the integration of interventions across community and hospital settings.^19^ Implementation science—defined as the scientific study of theories, models and frameworks to increase the uptake of evidence-based practices—can support the identification of multilevel contextual factors influencing the implementation of SCPs.^20^ These frameworks have been applied to various areas of survivorship care research, including examination of contextual factors^21 22^ (eg, partnerships and connections with community and academic organisations), and their impact on implementation outcomes (eg, intervention acceptability and feasibility).^23 24^

The purpose of our qualitative study was to explore PCPs’ experiences and perspectives related to the implementation of paediatric cancer SCPs within primary care clinics. The study was undertaken as part of an ongoing process evaluation of a multidisciplinary intervention, called ‘Engage’.^25 26^ Our study has four aims: (1) investigating PCPs’ preferences for the content and format of SCPs, (2) identifying implementation barriers and facilitators, (3) exploring preferences for educational resources and (4) examining attitudes toward shared-care approaches.

## Methods

This study was written in accordance with the Consolidated Criteria for Reporting Qualitative Research (COREQ) checklist (see online supplemental file 1).^27^

### Study context

The current study was conducted during a multi-site process evaluation and hybrid effectiveness-implementation trial of a paediatric brain cancer survivorship intervention, ‘Engage’.^25^ Briefly, ‘Engage’ is a remotely delivered, and multidisciplinary intervention that provides paediatric brain cancer survivors with personalised care recommendations, aiming to improve their health-related quality of life and self-efficacy.^25^ To participate in ‘Engage’ all survivors were required to provide the hospital survivorship clinic with a referral letter from their PCP. The development of the ‘Engage’ SCP, coordinated by the survivorship nurse, and a visual depiction of its contents are described in figure 1. The focus on brain cancer survivors in this study is due to their uniquely high and complex needs. If PCPs can manage some of the most medically complex survivorship care plans, then their perceived implementation challenges are likely addressable among other cancer diagnoses.

**Figure 1:**
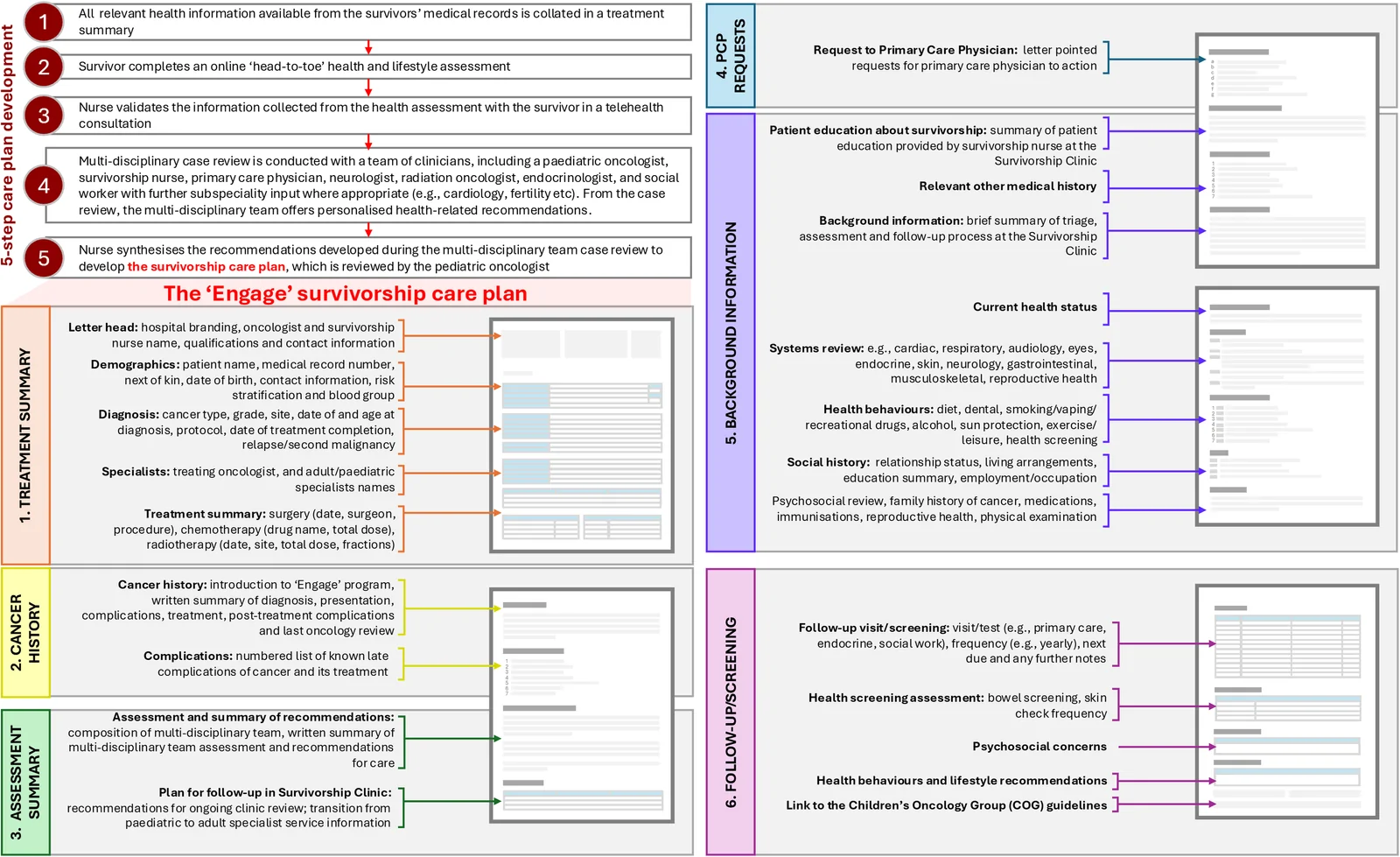
Development process, structure and content of the ‘Engage’ survivorship care plan. PCP, primary care physician.

### Recruitment and sampling

PCPs met inclusion criteria for this study if they were (1) currently practicing in Australia and (2) had an active medical registration as a specialist general practitioner (ie, the Australian regulatory designation; hereafter referred to as primary care physicians). Potentially eligible participants included PCPs who had referred patients to ‘Engage’ as well as PCPs from the general workforce (see figure 2 for recruitment procedure).

**Figure 2:**
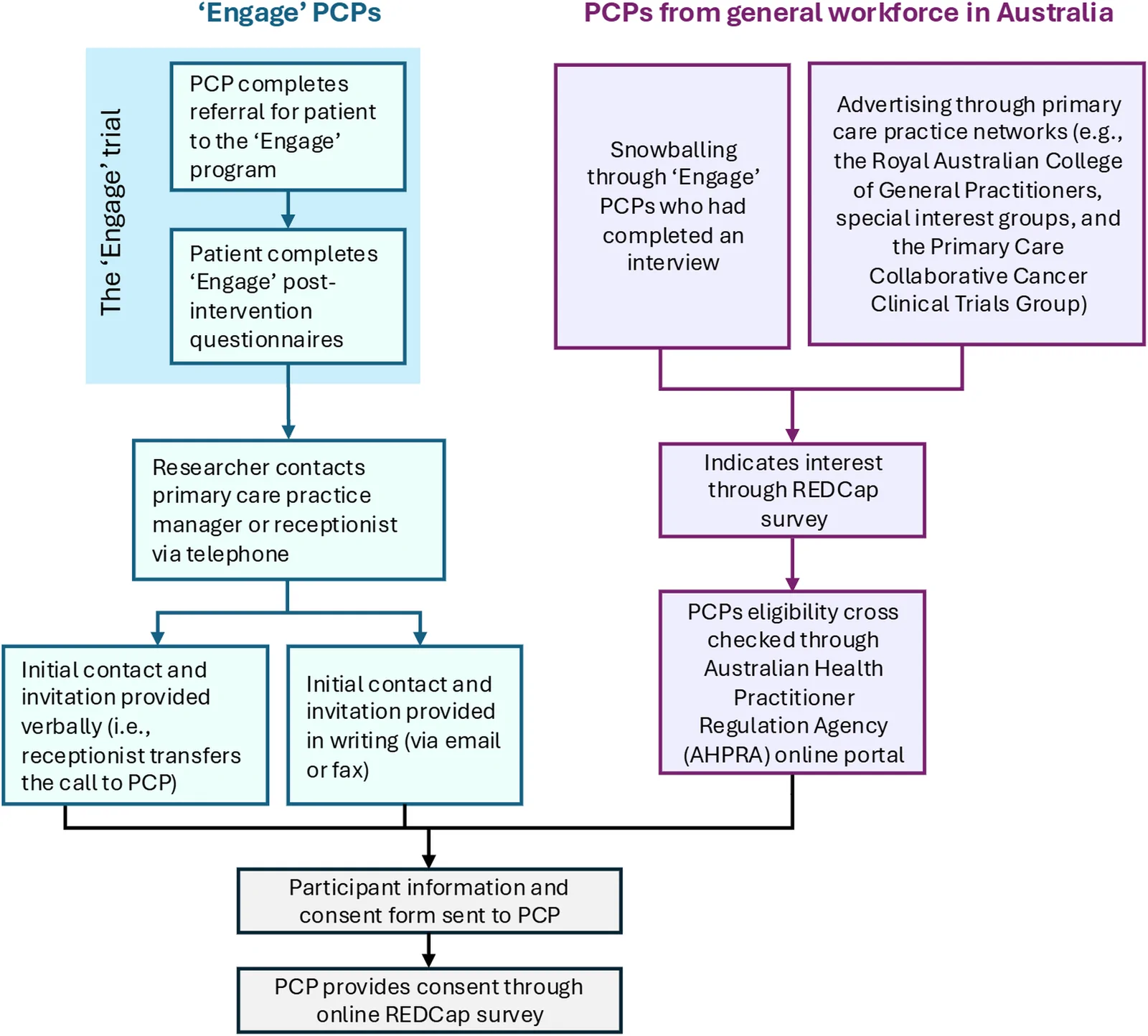
Flow diagram of participant recruitment procedure. PCP, primary care physician; REDCap, Research Electronic Data Capture.

All PCPs interviewed were emailed a copy of the participant information sheet and consent form. Consent was obtained both online via the survey platform REDCap and verbally at the commencement of each interview. ‘Engage’ PCPs were provided a de-identified copy of their patient’s SCP. All remaining PCPs were provided the same sample SCP, based on a real de-identified survivor, generated to guide conversation within this study. Interviews were conducted by an implementation science PhD Candidate (JE) via teleconferencing (ie, Zoom or Microsoft Teams) or telephone. All PCPs were provided with an $A200 e-gift card (~US$132) after completing the interview.

Sample size was guided by the principle of information power, which depends on factors such as study aim, sample specificity, use of theory, quality of dialogue and analytical strategy.^28^ Given the focused nature of our aims, the grounding of the study in implementation theory, the interviewer’s experience and the breadth of the interview schedule, we aimed to recruit 15 participants. The relevance, depth and variation of participants’ accounts in relation to the study aims, and the extent to which the data generated sufficient detail to support analysis, were discussed by the lead analyst (JE) and members of the study team (NT, JKM, CS, CM) at several points during recruitment and analysis. Recruitment ceased once sample adequacy was judged to have been reached, consistent with the principle of information power

### Theoretical orientation

The Consolidated Framework for Implementation Research (CFIR V.2.0) was selected due to its suitability for addressing all four research aims.^29^ The framework enables exploration of factors influencing the *Innovation* design (ie, survivorship care plans). It also enables identification of implementation factors at an organisational level, including the *Inner* (eg, within primary care practices) and *Outer settings* (eg, broader health system). In the *Individuals Domain*, the CFIR V.2.0 allows for further individual behaviour specification when mapped to the Theoretical Domains Framework (TDF).^30^ These frameworks can be used to develop evidence-informed recommendations through taxonomies such as the Expert Recommendations for Implementing Change (ERIC)^31^ and Behaviour Change Techniques (BCTs).^32^

### Data collection

The domains and constructs of the CFIR V.2.0 and TDF were used to provide an initial framework of the interview schedule (see online supplemental file 2). The schedule was then iteratively refined with two behavioural scientists (JKM and CS), two senior implementation scientists (NT and CM), two primary care physicians (ET-B and JR) and a paediatric oncologist (RJC). The schedule was organised into four sections aligning with our four study aims, (1) evaluating the SCP content and structure using a cognitive interviewing ‘think aloud’ exercise,^33^ (2) exploring factors influencing SCP implementation, (3) understanding preferences for informational supports and (4) exploring shared care. The schedule was piloted with two experienced PCPs, one who attended the remote survivorship clinic multidisciplinary team (MDT) review as part of formulating health recommendations for ‘Engage’ survivors (ET-B) and one external to the programme (RL).

### Data analysis

Interviews were transcribed verbatim and uploaded to NVivo V.14 for qualitative analysis.^34^ A mixed deductive-inductive approach was undertaken.^35^ First, two independent analysts (JE and SL) deductively coded 20% of transcripts to the domains and constructs of the CFIR V.2.0, which encompasses the TDF. A codebook was developed and agreed on by the two coders, which guided the remaining analysis by JE (see online supplemental file 3). This deductive approach, informed by principles of directed content analysis,^36^ was appropriate as it enabled the use of an existing framework to guide coding, and organise findings in a way that enhances their transferability to other contexts. Second, a thematic label was ascribed to group CFIR/TDF domains and constructs. This reflective thematic process was well suited to capture PCPs’ experiential insights of implementing care plans.^37^ Conflicts related to determinant coding and thematic labelling were resolved through discussions between the two analysts, with disagreements resolved with the wider team (JE, SL, JKM, CS, CM, NT). A conventional content analysis^36^ was undertaken for questions which explored PCPs’ preferences for developing educational resources. Categories for suggested format, topics and dissemination pathways were informed by the interview prompts, and allowed to emerge inductively, which were then quantified as response frequencies.

### Patient and public involvement

Patients were not involved in the conduct, reporting or dissemination of this study. However, individuals with lived experiences of cancer survivorship (parents, and a paediatric cancer survivor) have been involved as co-investigators in the broader conduct of the ‘Engage’ trial.

## Results

We identified 42 PCPs via the ‘Engage’ trial, of whom 37 (88%) did not respond to our invitation to participate. Of the 5 PCPs who responded, 2 opted out and 3 agreed to be interviewed. An additional 23 Australian PCPs, who were not involved in the ‘Engage’ programme, responded to our study advertisements of whom 15 ultimately participated, where enrolment was sequential based on the time of their response. Two PCPs were recruited via snowballing, resulting in a final sample of 20. Interviews ranged from 38 to 59 min (average 47 min) and were conducted between October 2023 and April 2024. 14 (70%) PCPs were female, had an average of 8.5 (range: 1.5–20) years of experience and 13 (65%) completed their primary medical training in Australia (see table 1).

**Table 1:** Participant characteristics

Characteristic	n (%) (N=20)
Sex	
Female	14 (70)
Male	6 (30)
Mode	
Teleconferencing (with video)	13 (65)
Phone	7 (35)
Recruitment path	
Primary care practice networks	14 (70)
’Engage’ intervention	3 (15)
Snowballing	2 (10)
Social media	1 (5)
Country of primary medical training	
Australia	13 (65)
Iran	2 (10)
Hong Kong SAR, China	1 (5)
China	1 (5)
Greece	1 (5)
Nepal	1 (5)
UK	1 (5)
Practice state	
New South Wales	11 (55)
Victoria	3 (15)
Western Australia	2 (10)
South Australia	1 (5)
Queensland	1 (5)
Tasmania	1 (5)
Australian Capital Territory	1 (5)
Remoteness classification of primary practice^*^	
Metropolitan areas	15 (75)
Large rural towns	4 (20)
Regional centres	1 (5)
Practice type	
Private	18 (90)
Community	2 (10)
Usual billing arrangement	
Private billing	8 (40)
Mixed billing	7 (35)
Public billing	5 (25)
Estimated survivors cared for in prior 2-year period	
0	3 (15)
1–2	9 (45)
3–4	4 (20)
5+	4 (20)
Interview sections completed:	
‘Think aloud’ letter evaluation.	13 (65)^†^
Implementing the survivorship plan	20 (100)
Educational and informational supports.	20 (100)
Shared model of survivorship care.	5 (25)

* Based on the MMM classification.

† Denotes participants who completed the ‘think aloud’ exercise, though 18 (90%) of participants provided substantive feedback about the SCP.

MMM, Modified Monash Model; SAR, Special Administrative Region; SCP, Survivorship Care Plan.

Data were analysed according to SCP content, implementation factors, educational needs and models of care. Further constructive feedback is detailed below, and recommendations for the implementation and development of an optimal SCP are summarised in figure 3 (see online supplemental file 4 for links between recommendations to theoretical constructs and evidence-informed strategy taxonomies, and online supplemental file 5 for a revised version of figure 1 illustrating how the SCP could be amended). Findings below reflect both PCPs’ anticipated implementation experience based on review of the same sample survivorship care plan (17/20), and direct/actual implementation experience (3/20). The three 'Engage’ PCPs and general workforce PCPs were analysed together given the small number in the former group and relevant subgroup denominators are reported throughout to aid interpretability.

**Figure 3:**
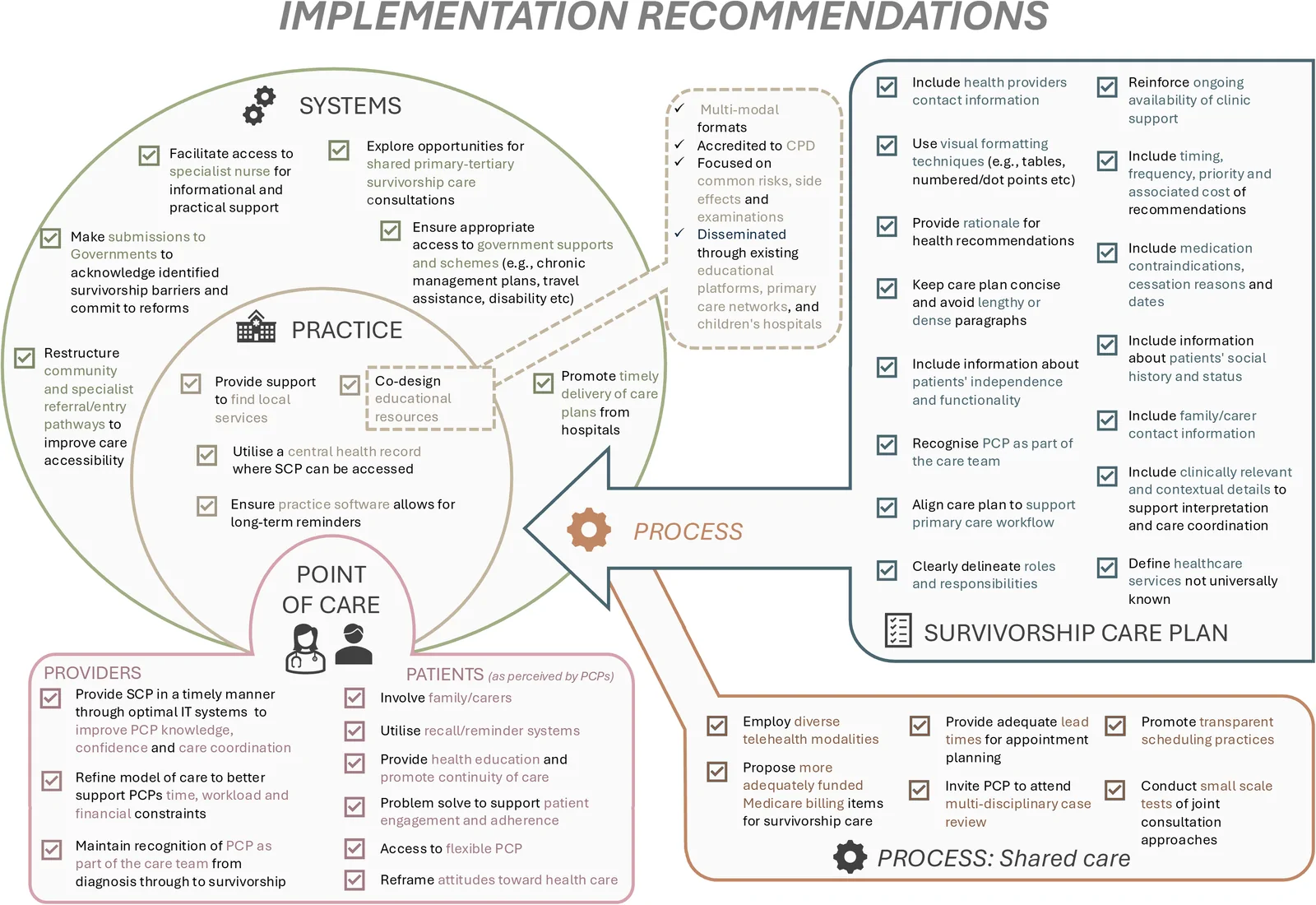
Summary of our recommendations for implementing survivorship care plans. CPD, Continuing Professional Development; IT, information technology; PCP, primary care physician; SCP, Survivorship Care Plan.

### SCP content and format (CFIR domain, innovation)

Overarching criticisms of the SCP included the use of unexplained abbreviated terms, information repetition across sections and dense/lengthy paragraphs. Improved formatting was thought to improve the salience of pertinent information, especially given that PCPs only briefly scanned the SCP prior to their consult. PCPs also wanted a separate section on the patient’s social functionality and level of independence. Online supplemental file 6 provides a summary of PCPs’ attitudes toward the SCP and suggested adaptations. Quotes were selected to illustrate some of the key features discussed and adaptations proposed.

#### Treatment summary

Several PCPs described information about treatment dosages as not in their scope of practice. PCPs suggested including only the treatment name and dates administered to qualify whether it was a high/low dose or include this information in an appendix. Most PCPs appreciated inclusion of the names and contact details of survivors’ cancer specialists. Survivors were assigned a risk stratification score from 1 to 3, indicating how they were triaged based on a risk assessment of their diagnosis and treatment exposures. PCPs found the risk stratification score unclear and recommended qualifying its meaning.

#### History

Several PCPs found the history section to be useful, though one felt that it was ‘too much information’ noting that the specifics of the hospital procedures were not as relevant. Many PCPs found the dot-pointed list of known late complications from cancer and its treatment particularly helpful, noting that patients are not always fully aware of their late effects, or if cancer is the cause of a current illness. Several PCPs indicated that dates of complications should be provided in brackets next to this list.

#### Assessment

PCPs valued knowing which clinicians/roles were involved in the MDT. One PCP found the suggestions to improve the survivors’ lifestyle and social behaviours as redundant, as they believed no health practitioner would advise otherwise. PCPs described the table summarising their transition plan from paediatric to adult specialists as useful, noting it would be especially helpful for early career providers who may be less familiar with appropriate specialists for referral. Several participants also emphasised the importance of including details about intracranial-shunt management—specifically, which provider was responsible for its management and how the family would support care.

#### PCP requests

PCPs appreciated dot point formatting for actions they were expected to implement, finding them concise, easy to follow and clearly itemised. PCPs preferred their placement at the start/end of the care plan rather than the middle. PCPs criticised the lack of rationale for why certain items had been requested and often reported that the language used failed to convey survivors’ specific needs, including the timing, frequency and method of recommendations. For example, when screening measures were requested (eg, bowel cancer), some PCPs desired further clarification on whether the screening method differed from routine population-based approaches. One PCP expressed confusion about the term ‘ongoing specialist referrals’, explaining that such requests are redundant unless their patient’s condition changes or a new issue arises. Others were unsure which requests to prioritise first in their consult and suggested to order them by priority. PCPs also raised concerns about the tone used in the requests, expressing a desire to be more formally acknowledged as part of the care team and thanked for their role in implementing the care plan.

#### Background

PCPs highlighted the patient’s social and psychosocial information as particularly valuable, especially for understanding patients’ support systems, but suggested restructuring the *background section* to serve as an executive summary/action plan with detailed information moved to an appendix. They also recommended clearer mental health indicators, expanded medication details (eg, contraindications, cessation dates and reasons for discontinuation) and better insight into patients’ current functioning (eg, impact of cranial nerve palsies on vision and daily activities like driving).

#### Follow-up/screening

PCPs found the initial follow-up visit summary table useful for quickly discerning the roles of all health professionals involved in the survivors’ care, and some found that the layout supported their process of inputting reminders/recalls into their practice software. Several PCPs expressed a desire for greater specificity in the follow-up recommendations provided, describing phrases such as ‘as per [speciality]’ as vague and of limited practical value. Broad instructions for the PCPs to conduct a ‘comprehensive history and physical examination’ were viewed as unclear and several preferred more specific guidance. PCPs also desired to have their role and assigned tasks in the follow-up table made more explicit, and suggested ordering rows by priority (eg, by time next due and clinical urgency). Regarding the second cancer screening table, PCPs valued the links to screening guidelines, however they recommended indicating potential out-of-pocket costs when surveillance deviated from normal population guidelines which are funded by public health programmes.

### Factors influencing implementation (CFIR domains, inner/outer setting and individuals)

Factors influencing the implementation of SCPs were organised into four themes including: structural and systems related factors, practice-level factors, provider-level factors and patient level factors. A summary of illustrative quotes and their respective subthemes are provided in table 2, with the complete set provided in online supplemental file 7.

**Table 2:** Themes, CFIR V.2.0/TDF constructs and a selection of illustrative quotes related to survivorship care plan implementation

Subtheme: Barrier (−), facilitator (+)	Framework, construct	Illustrative quote (participant demographics)
Theme 1: structural and systems level factors (outer setting)		
Access to community care and specialist resources (−)	CFIR, local conditions	“I’ve got patients who will die of bowel cancer before they get the scope because there’s a two to three year waiting list, even if they have bowel cancer symptoms and positive for blood tests…there’s no psychology access at all… it’s so frustrating trying to get people to see specialists they need to see …” (female, metropolitan, 3 years’ experience)
Communications with hospitals/specialists (−)	CFIR, partnerships and connections	“The rejection of the referrals is a massive phenomenon in my area, because I understand [hospitals are] struggling. They don’t have the capacity, [and] letters are being missed. We’re using faxing, like we’re in 1940s or 50s, instead of streamlining the transfer of information…I’ll wait two or three weeks to receive the letter…. So it takes a number of weeks for the admin for the letters to be processed, not from my side, but from the hospital…I think the whole communication between primary care and secondary care should improve” (male, metropolitan, 6 years’ experience)
Patient advocacy and leveraging relationships with clinicians (+)	CFIR, partnerships and connections	“I’ve sort of made connections with colleagues on the way, and so if something needs to happen urgently, I’ll just send them a text and say, hey, can you help me sort this patient out? Depending on what it is, and usually that will happen” (male, metropolitan, 8 years’ experience)
Access to survivorship nurse (+)	CFIR, partnerships and connections	“…typically it’s always the [nurses] who are just like guns and so on the ball and super awesome in getting back to you…just having a number or an email address is super handy” (female, metropolitan, 3 years’ experience)
Ongoing eligibility for PCP management plans (+)	CFIR, policies and laws	“…patients that can access our clinic, we can help them to fund the care through a chronic disease plan. It gives us regular reviews, which lets us chip away at these things. Some things won’t have to be done right on the first day that they come in. This is a kind of annual plan, and some things might be even biannual. So, the clinic’s accessible, the appointments are accessible, the costs are manageable” (female, metropolitan, 15 years’ experience)
Theme 2: practice-level factors (inner setting)		
Practice software constraints (−)	CFIR, information-technology infrastructure	“…the problem with our practice software, you can’t actually put in a recurring reminder, which would be very useful…when something like [this health recommendation] comes, we can put in a reminder for the next one. So say, in a year’s time, needs a skin check, needs a full medical, but then once that comes around and that reminder is actioned, then we have to put in another reminder for the year after” (male, metropolitan, 13 years’ experience)
Centralised health record (+)	CFIR, information- technology infrastructure	“[We] encourage young person’s to be registered with MyHealth record. So in that case every health professional can access, and you upload it into MyHealth record and with young person’s permission, we all can access the detailed information. So that’s very important, especially young person can be very mobile, not necessarily stay in one location for very long. If they move to a different location, they will require a new GP” (female, metropolitan, 15 years’ experience)
Theme 3: provider-level factors (individuals, roles: innovation deliverers)		
Time constraints and pressures (−)	CFIR, opportunity, TDF, environmental context and resources	“I guess the way that my practice is done, which is bulk billing, means that you only have limited time to do limited things. And I guess my concern would be that, and this is speaking very frankly and honestly, a yearly thyroid palpitation, I don’t think that will ever be done, and because that’s not something that I would do unless (a), the patient prompts me, or (b) it’s something that is part of something that I normally do anyway” (male, metropolitan, 8 years’ experience)
Knowledge of survivorship care (−)	CFIR, capability, TDF, knowledge	“Yeah, it’s a very foreign term especially GPs who are roughly around the same stage that I am at. I don’t think a lot of us have ever heard the word survivorship, or cancer survivorship before” (female, metropolitan, 8 years’ experience)
Improved confidence through care plans (+)	CFIR, motivation, TDF, beliefs about capabilities	“…it’s very helpful to have a list of the requirements, the appointments, follow up screening, and you know which ones we are to organise and which ones we need to refer to other specialties to provide. So that would definitely increase my confidence in terms of managing these patients appropriately” (male, metropolitan, 8 years’ experience)
Improved care coordination through care plans (+)	CFIR, partnerships and connections	“At the moment and how it has been for a long time, there is this big segregation between public hospital and general practices. They say that we’re together, but we’re not really together because often there is this lapse of information and we’re often not included in the treatment plan. We often don’t know what is happening when they review patients in their consultation rooms…So having such a detailed letter is the dream because then I feel like I am part of that team. I feel like I respected and having an active role in this patient’s life…” (female, metropolitan, 6 years’ experience)
Theme 4: patient-level factors (individuals, roles: innovation recipients)		
Financial capacity (−)	CFIR, opportunity, TDF, environmental context and resources	“We do see quite a few struggling parents that may have to save up and wait for pay to come in before they can actually see a specialist. And we’ve had patients having to reschedule cancer appointments because they just haven’t got the money to see the specialist on that day.” (male, metropolitan, 8 years’ experience)
Access to services for rural and remote patients (−)	CFIR, opportunity, TDF, environmental context and resources	“…most of the time they need to get like a surgery, stuff like that, or even some of the rehab services is not available here. So, travelling to [Metropolitan Hospital 1] is two hours away from us. [Metropolitan Hospital 2] is at least four hours from us. And the close hospital that will be by plane. So, this is something that we are now facing and struggling with.” (female, rural, 2 years experience)
Young people avoidance (−)	CFIR, motivation, TDF, emotion	“…adolescents are notoriously bad at engaging with healthcare because they just want to be kids and enjoy their lives” (female, rural, 11 years’ experience)
Family/carer support (+)	CFIR, opportunity, TDF, social influence	“A good support network at home. So family or close friends…Just to pick you up when you are down, go for a walk, keep doing everything, you’re doing encouragement.” (female, metropolitan, 4 years’ experience)
Strategies to support engagement/adherence	CFIR, engaging, innovation recipients	“…when they come for medications, we’ve been trained… [to say] oh, by the way, do you know you’re due for this? So, yes, you’re right, it can be done opportunistically. So, when they come for medication, so if you attach it to something that they’re due for then it’s much easier to get them to come, get them returning…” (female, metropolitan, 2 years’ experience)

CFIR, Consolidated Framework for Implementation Research; GP, general practitioner; PCP, primary care physician; TDF, Theoretical Domains Framework.

#### Theme 1: Structural and systems related factors influencing implementation of recommendations made in the care plan (outer setting)

Access to community-based services (including allied health and psychosocial supports), and specialist medical services (eg, endocrinology, neurology, ophthalmology) emerged as the most prominent barrier (*CFIR, Local Conditions),* though differed depending on the state of practice, proximity to a hospital and the type of health service sought. Common challenges included limited-service availability resulting in long wait times and referral rejections (see online supplemental file 7, quotes 1a–b). Notably, all rural and remote PCPs (4/4) reported difficulties facilitating access to specialist and/or community services for their patients (quotes 1c–e). Communication with hospital specialists was also a significant challenge (*CFIR, Partnerships and Connections*). PCPs often relied on communication given by hospital switchboards or on-call registrars, who lacked patient-specific knowledge. Delays in receiving care plans or correspondence also contributed to poor communication (quote 1f–g). While private specialists were generally perceived as more accessible, wait times and cost often constrained patient access to care. To overcome these barriers, several PCPs described drawing on personal networks or persistently advocating for their patients to be seen (*CFIR, Partnerships and Connections*) (quotes 1h–i).

The most commonly reported facilitator to implementing the recommendations made in the SCP was having access to the survivorship nurse at the hospital clinic (*CFIR, Partnerships and Connections*) (quotes 1j–k). PCPs believed that access to a specialist nurse provided a valuable point of contact after receiving the SCP—particularly when navigating investigations outside standard guidelines, interpreting abnormal results, managing acute issues or identifying appropriate local referrals. PCPs believed this also offered opportunities for education in complex areas of care and avoided defaulting to emergency services when urgent specialist input was needed. Several PCPs drew on previous positive experiences with specialist nurses in other disease areas to support this view. Ongoing eligibility for PCP management plans (ie, a subsidised primary care item under the Australian national health insurance scheme, Medicare) was also seen as a facilitator, enabling structured, low-cost and regular reviews to support long-term chronic healthcare management (*CFIR, Policies and Laws*) (quote 1l).

#### Theme 2: Practice level factors influencing implementation (inner setting)

PCPs identified software constraints to implementing survivorship care recommendations (*CFIR, Information-Technology Infrastructure*), noting difficulties in generating long-term recalls and reminders due to limited scheduling capabilities (quote 2a). They also highlighted the potential role of centralised electronic health records (eg, Australian Government’s MyHealth Record) for storing SCPs, improving accessibility and provider awareness, while also supporting continuity of care in primary practice (quote 2b).

#### Theme 3: Provider level factors influencing implementation (individuals, roles: innovation deliverers)

The most significant provider level barrier included significant workload pressures, time constraints and financial disincentives imposed by publicly funded Medicare items (*CFIR, Opportunity, TDF, Environmental Context and Resources*). PCPs characterised these factors as limiting the feasibility of facilitating survivorship care recommendations (quotes 3a–b). Gaps in PCP knowledge of survivorship care were also noted (*CFIR, Capability, TDF, Knowledge*) (quotes 3c–d). SCPs themselves were perceived as facilitators that could improve their confidence to provide care (*CFIR, Motivation, TDF, Beliefs About Capabilities*) (quotes 3e–f), and improve care coordination with the survivorship clinic, and hospital specialists (*CFIR, Partnerships and Connections*) (quote 3g).

#### Theme 4: Patient level factors influencing implementation (individuals, roles: innovation recipients)

PCPs reported that patient level factors related to difficulties engaging survivors and promoting their adherence to survivorship recommendations. Key challenges included the financial burden associated with adhering to recommendations (quote 4a) and limited access to services for individuals living rurally/remotely (quote 4b) (*CFIR, Opportunity, TDF, Environmental Context and Resources*). PCPs also described avoidance behaviours, with young people perceived as not interested in seeing their PCP and engaging with lifestyle and health behaviour recommendations (*CFIR, Motivation, TDF, Emotion*). Disengagement from cancer-related care was often perceived as attributable to survivors’ focus on resuming routines and activities typical of their peers (quote 4c). PCPs also described healthcare fatigue following acute care and follow-up, as well as fear or aversion stemming from traumatic experiences of receiving cancer care (quotes 4d–e). These factors were perceived to disrupt continuity with primary care and reduce the likelihood of sustained engagement (quote 4f). Facilitators included social and motivational support from family/carers (*CFIR, Motivation, TDF, Social Influence*) and continuity with a skilled, supportive PCP (quote 4g–h). Additional strategies involved using recall and reminder systems (quote 4i), providing health education to explain the risks associated with disengagement and the importance of the PCP’s role (quotes 4j–k) and taking an opportunistic approach when survivors presented to them for unrelated concerns (quote 4l) (*CFIR, Engaging - Innovation Recipients*).

### Educational and informational supports (CFIR, inner setting, access to knowledge and information)

Most participants (n=18, 90%) reported that educational resources would be ‘helpful’ to support survivorship care delivery (see table 3). PCPs endorsed a range of formats, most commonly brief written summaries (n=9; 45%) and short videos (n=7; 35%), with some noting that multimodal materials would be preferential to accommodate the wide cast of learning styles. Several PCPs also recommended that educational resources be accredited to Continuing Professional Development (CPD) points.

**Table 3:** Primary care physicians’ preferences for childhood cancer survivorship educational resources

Variable	Frequency n, (%) (n=20)
Preferred formats	
Brief written summaries	9 (45)
Short videos	7 (35)
CPD accredited	6 (30)
Checklists	3 (15)
Webinar or lecture	3 (15)
Multimodal formats	2 (10)
Patient resources	1 (5)
Annual conference	1 (5)
Observe specialist consultations	1 (5)
Podcasts	1 (5)
Topic areas	
Common risks/side effects/complications and examinations	14 (70)
Screenings/blood tests	4 (20)
Secondary cancers	4 (20)
Mental health	3 (15)
Endocrine	2 (10)
Learning and social development	2 (10)
Sexuality and intimacy	1 (5)
Neurology	1 (5)
Cognitive	1 (5)
Swallowing difficulties	1 (5)
Hearing and balance	1 (5)
Preventative healthcare	1 (5)
Cardiology	1 (5)
Sleep	1 (5)
Holistic overview of survivorship care	3 (15)
Checklist for annual survivorship appointment	1 (5)
Local services for referral	1 (5)
Appointment planning	1 (5)
Summary of Clinical Oncology Group guidelines	1 (5)
PCP management plan template	1 (5)
Preferred dissemination pathways	
Online dedicated primary care local health information and decision support portal (ie, HealthPathways)	12 (60)
Central/largest professional primary care organisation in Australia (RACGP, hosted on the organisations website or via newsletter/emails)	9 (45)
Children’s hospital website	6 (30)
Contained within SCP	4 (20)
Primary healthcare networks	2 (10)
Email directly	1 (5)

CPD, Continuing Professional Development; PCP, primary care physician; RACGP, Royal Australian College of General Practitioners; SCP, Survivorship Care Plan.

### Shared model of survivorship care (CFIR domain, process)

Some PCPs were asked about preferences for a joint primary-tertiary consultation to discuss the SCP (n=5, including one who had a patient participate in ‘Engage’), and each responded positively to this concept. This small subset of PCPs perceived that a joint consult would improve shared understanding of roles and responsibilities and provide an opportunity to ask questions (*CFIR, Teaming*). Notable challenges to implementing this model included ensuring the joint consult is publicly billed (ie, through a Medicare item) (*CFIR, Policies and Laws*) and is appropriately aligned with PCPs availability particularly given that in general practice, providers are often late to their scheduled appointment times (*CFIR, Work Infrastructure*). Suggested ways to further operationalise the approach (*CFIR, Adapting*) included employing diverse telehealth modalities (ie, phone, digital platforms) and providing adequate lead times to plan for appointments.

## Discussion

PCPs highlighted their preferences for concise, well organised and clinically relevant SCPs. They identified several areas for improvement to content and structure, which would better support their consultations and help them understand their responsibilities compared with tertiary and secondary providers. PCPs reported several key challenges influencing SCP implementation including accessing community and specialist health services, communicating with hospitals, funding, insurance and remuneration schemes and maintaining continuity of care with survivors. PCPs also highlighted several facilitators including having access to a hospital-based nurse specialist for informational support and desires for tailored educational resources. A subset of PCPs (5/20) asked about shared care expressed positive attitudes towards joint consultative approaches, though perceived difficulties with its operationalisation. Collectively, our findings informed recommendations for developing and implementing SCPs in practice (see figure 3), with a selection of evidence-informed recommendations denoted in brackets in our discussion (ie, ERIC/BCT). By interrogating multilevel implementation factors, we move the field of survivorship care implementation beyond describing what stakeholders think about SCPs toward clarifying the implementation conditions influencing adoption and delivery in practice.

PCPs preferred SCPs that were compact, easily actionable, well-structured and easy to process quickly, with visual cues to enhance salience. Concerns about overly complex care plans have been voiced in the literature,^32 38^ highlighting the ongoing challenge of balancing concise, high-level summaries while ensuring access to detailed information to support complex medical care and accommodate varied PCP preferences. Additionally, childhood cancer survivors, especially those who have survived a brain cancer malignancy, are likely to have multifaceted, complex and sometimes lengthy needs, which presents another tension to balancing the breadth and depth of information required to facilitate effective care. It is also important to recognise that highly specialised information within the care plan may be relevant to other healthcare professionals involved in the survivor’s care (eg, a cardiologist would need to know the amount of potentially cardiotoxic treatment received). A potential solution suggested was to include an executive summary within the SCP, tailored to support the care coordination role of PCPs.

Given the time constraints reported in both our study and elsewhere,^39^ PCPs emphasised the importance of clearly prioritising urgent follow-up actions. These findings highlight a broader challenge in primary care of reconciling underfunded consultation times with delivering complex, long-term chronic healthcare. Design features such as tables, dot points, utilising different colours, bolding and underlining were proposed to improve the efficiency of reviewing care plans (*ERIC: promote adaptability*). PCPs recommended including contact details for involved subspecialists (eg, endocrinology, neurology) reflecting their responsibility for care coordination and emphasised the need for role differentiation from other members of the multidisciplinary team. Delineation of roles has been proffered as challenges faced by PCPs delivering survivorship care.^38 40^ PCPs also called for more information related to survivors’ social history, functioning and level of independence. Taken together, our findings raise important questions about whether hospital-developed care plans currently align with the realities and priorities of primary care, where physicians need concise, practical information to support community management rather than extensive specialist-focused details.

PCPs are well positioned to care for many survivors, providing more accessible, cost-effective and holistic care as they live, study and work within their community. However, PCPs in this study and elsewhere have voiced their preference for caring for survivors in consultation with survivorship clinics, and report discomfort when expected to manage survivors without points of contact from the cancer specialist team.^32 40 41^ Hospital-based specialist nurses can provide a critical point of contact for PCPs to navigate care for their patients.^42 43^ This is essential, as even with targeted education, PCPs will continue to require access to cancer specialist expertise for referral support and timely tertiary intervention when late effects emerge or worsen. In publicly funded settings with finite cancer specialist capacity, reserving tertiary survivorship resources for higher-risk or complex survivors, combined with a dedicated nurse liaison for PCPs, may similarly improve system efficiency. We therefore recommend hospitals prioritise formalising structured advice and escalation pathways (eg, a named survivorship nurse liaison) that PCPs are aware of and can use for support (eg, contact advice provided within the SCP, or searchable through primary health platforms) (*ERIC: create a learning collaborative*).

PCPs in our study highlighted difficulties accessing public health services. These challenges are compounded by the already significant time and workload pressures PCPs face,^44^ with care often delivered reactively in response to urgent needs, leaving little capacity for proactive follow-up planning. These concerns were pronounced among rural and remote PCPs in our sample, all of whom reported barriers to accessing community and specialist services. Geographical disparities in access to optimal survivorship care are well documented,^45^ with resource-limited settings placing greater responsibility on PCPs to deliver survivorship care.^38^ Public hospitals play an important role in enabling survivors to re-access specialist survivorship outpatient services when needs emerge following transition to primary care (*ERIC: promote network weaving*). A systems-level solution may be to establish a formal re-entry pathway, enabling PCPs to more easily refer survivors back to survivorship specialist review when clinically indicated (*ERIC: promote adaptability*). Clarity around this pathway is particularly important for long-term survivors who have aged out of the paediatric system in the interim between acute cancer care and the development of late effects.

### Developing educational and information supports

Most PCPs (18/20) supported the development of primary care tailored educational resources in survivorship care. Barriers to implementing SCPs are often framed around PCPs’ limited knowledge and confidence, shaped by gaps in survivorship expertise, the absence of well-defined primary care guidelines and insufficient training opportunities in survivorship care.^41^ While targeted education can increase survivorship knowledge^41^ (*BCTs: instruction on how to perform behaviour*), primary care often operates within a time-poor setting, which makes realising broad educational approaches for rare diseases challenging. One key tension is the unrealistic expectation that PCPs acquire and maintain the breadth of specialised survivorship knowledge (eg, the Children’s Oncology Group Guidelines exceed 200 pages). Another is a misalignment between guidelines designed for cancer specialist-led contexts, and the practical needs of primary care, where concise resources are preferred. These challenges mirror broader primary care implementation research, in which clinicians have generally been receptive to interventions that address knowledge gaps, yet have opposed those that exacerbate existing organisational and external-system constraints (eg, insurance and regulatory pressures) even when supportive of the underlying goals.^46^ Therefore, we propose developing targeted educational resources (eg, designed for PCPs currently caring for a childhood cancer survivor), that are low-burden, incentivised^38^ (eg, CPD accredited), and co-designed with PCPs to improve uptake (*ERIC: develop/distribute educational materials*).

### Models of shared survivorship care

While our findings validate interest from a small subset of PCPs’ (5/20) to be more integrated in the transition process from hospital to primary care led follow-up,^47^ information technology and health systems factors may inhibit acceptability and adoption. Recent evidence describing an oncology primary care clinic embedded within a tertiary cancer centre provides one example of how oncology–primary care integration can be operationalised in practice. The model included survivorship-informed primary care, co-location with oncology services, multidisciplinary coordination and a patient outcomes registry to support evaluation.^48^ Telehealth offers a mechanism to support feasible joint consultations (*ERIC: change physical structure and equipment*),^38^ however implementation challenges include PCPs’ consultation availabilities, and inter-organisation scheduling practices. Given the unpredictable time-pressured nature of both hospital and primary care settings, PCPs expected that one or more parties would run late. Shared care may also necessitate restructuring Medicare and insurance billing arrangements (*ERIC: make billing easier*). In Australia, Medicare is the national universal health insurance scheme that includes subsidised access to primary care services. Existing chronic condition management pathways within Medicare only offer limited additional support for care coordination and access to rebateable allied health services. Medicare rebates for longer PCP consultations are also lower on a per-minute basis than those for shorter consultations, which devalues appointments for patients with complex health needs like childhood cancer survivors.^49^ One solution is to propose dedicated Medicare, Medicaid or insurance benefit scheme item numbers that reflect the time and complexity required to deliver survivorship care.

### Strengths and limitations

Although male PCPs were under-represented, our sample included PCPs across diverse settings, including from all but one Australian state/territory, across rural/regional areas and with varied billing practices, capturing a wide range of perspectives. While the Australian primary care context has distinctive features the findings identified in this study, such as time constraints, role delineation and the value of specialist nurse support, are likely to resonate across health systems with similar primary-specialist care interfaces. Most PCPs (n=17, 85%) reviewed the same ‘Engage’ care plan, limiting their frame of reference to a single brain cancer survivors’ case, which may constrain generalisability to other cancer diagnoses. However, survivors of brain malignancies often have more complex care needs compared with other survivor cohorts,^50^ likely reflecting a more conservative estimate of SCP feasibility and usability in primary care.

Another limitation was the low response rate among PCPs nominated by ‘Engage’ participants (overall participation: 3/42, 7%). For comparison, PCPs participation in qualitative survivorship research can range from 46% to 88%.^6 13 41 42^ Our low response rate (~7%) may be influenced by commonly reported barriers in primary care research, such as time and scheduling constraints or level of interest in the research topic. Additionally, the sample size of PCPs asked about shared care was small (5/20, 25%), as these questions were not a core focus across all interviews. The uneven sample distribution between ‘Engage’ PCPs and those from the general workforce constrained our capacity to make distinctions between findings that reflect real-world use via the ‘Engage’ sample compared with those that reflect anticipated implementation. This also reflects a broader methodological tension between the study’s objective, which was to explore PCPs’ experiences implementing the ‘Engage’ survivorship care plan, and the composition of the sample, where the majority of PCPs (17/20) reviewed the care plan for the first time during or shortly before the interview. The ‘think-aloud’ exercise provided a robust approach that potentially allowed PCPs to imagine reviewing this care plan within the context of their own practice, lending ecological validity to their responses.

## Conclusion

Our qualitative study identifies key gaps in how SCPs are implemented in primary care and provides practical recommendations to ensure that care plans are clear, comprehensive, well-structured and appropriately tailored. Without careful design and implementation, care plans alone may be insufficient to improve the effectiveness of primary care led survivorship care. Further engagement with PCPs is essential to build capacity and support the ongoing development and evaluation of shared care models which include resources such as SCPs.

## Supplementary material

10.1136/fmch-2026-004002 online supplemental file 1

10.1136/fmch-2026-004002 online supplemental file 2

10.1136/fmch-2026-004002 online supplemental file 3

10.1136/fmch-2026-004002 online supplemental file 4

10.1136/fmch-2026-004002 online supplemental file 5

10.1136/fmch-2026-004002 online supplemental file 6

10.1136/fmch-2026-004002 online supplemental file 7

## Data Availability

Data are available upon reasonable request. All data relevant to the study are included in the article or uploaded as supplementary information.
