# Supplementary material for: Implementing childhood cancer survivorship care plans: qualitative perspectives and experiences from Australian primary care physicians
Source: Fam Med Community Health. 2026 Nov 5;14(Suppl 1):e004002. doi: 10.1136/fmch-2026-004002 (PMC13756439; doi:10.1136/fmch-2026-004002)
Supplement: Supplementary file 1 [file fmch-14-Suppl_1-s004.pdf]

Supplementary file 4. Alignment of recommendations to the Behaviour Change Technique (BCT) and Expert Recommendations for Implementing Change (ERIC) frameworks

Figure 3. Development		Evidence		Strategy development or alignment using theoretical framework				
				Recommendation development process				
Level	Recommendation	Evidence source	Related barrier/facilitator (if applicable)	*Evidence-informed*	*Thematic/ intuitive*	Determinant	Selected strategy taxonomy	Evidence-informed/aligned strategy
Systems	Restructure community and specialist referral/entry pathways to improve care accessibility	Barrier identified	Access to community care and specialist resources	✓		CFIR, Local conditions	ERIC	Promote network weaving
	Make submissions to Governments to acknowledge identified survivorship barriers and commit to reforms	Barrier identified	Access to community care and specialist resources; Ongoing eligibility for PCP management plans	✓		CFIR, Policies & laws, Local conditions	ERIC	Conduct local needs assessment
	Facilitate access to specialist nurse for informational and practical support	Facilitator identified	Access to survivorship nurse		✓	CFIR, Partnerships and connections	ERIC	Capture and share local knowledge
	Ensure appropriate access to government supports and schemes (e.g., chronic management plans, travel assistance, disability etc)	Barrier identified	Patients' financial capacity; Access to services for rural and remote patients	✓		TDF, Environmental context and resources (individual)	BCT	Problem solving
		Facilitator identified	Ongoing eligibility for PCP management plans		✓	CFIR, Policies and laws (organisational)	ERIC	Make billing easier
	Promote timely delivery of care plans from hospitals	Barrier identified	Communications with hospitals/ specialists	✓		CFIR, Partnerships and connections	ERIC	Promote network weaving
Practice	Ensure practice software allows for long-term reminders	Barrier identified	Practice software constraints	✓		CFIR, Structural characteristics –	ERIC	Change physical structure and equipment

Figure 3. Development		Evidence		Strategy development or alignment using theoretical framework				
				Recommendation development process				
Level	Recommendation	Evidence source	Related barrier/facilitator (if applicable)	*Evidence-informed*	*Thematic/ intuitive*	Determinant	Selected strategy taxonomy	Evidence-informed/aligned strategy
						information-technology infrastructure		
	Provide support to find local services	Facilitator identified	Access to survivorship nurse		✓	CFIR, Partnerships and connections	ERIC	Capture and share local knowledge
	Utilise a central health record where SCP can be accessed	Facilitator identified	Centralised health record		✓	CFIR, Structural characteristics – information-technology infrastructure	ERIC	Capture and share local knowledge
	Co-design educational resources<br>• Multi-modal formats<br>• Accredited to CPD<br>• Focused on common risks, side effects and examinations<br>• Disseminated through existing educational platforms, primary care networks, and children's hospitals	Content analysis	Preferences for mode, topics, and dissemination pathways for educational resources		✓	CFIR, Access to knowledge and information	ERIC	Develop and disseminate educational materials
Point of care	Provide SCP in a timely manner through optimal IT systems to improve PCP knowledge, confidence and care coordination	Facilitator identified	Improved confidence through care plans		✓	TDF, Beliefs about capabilities	BCT	• Information about health consequences<br>• Salience of consequences<br>• Information about social and environmental consequences

Figure 3. Development		Evidence		Strategy development or alignment using theoretical framework				
				Recommendation development process				
Level	Recommendation	Evidence source	Related barrier/facilitator (if applicable)	*Evidence-informed*	*Thematic/ intuitive*	Determinant	Selected strategy taxonomy	Evidence-informed/aligned strategy
								• Information about emotional consequences
	Refine model of care to better support PCPs time, workload and financial constraints	Barrier identified	Time constraints and pressures		✓	TDF, Environmental context and resources	BCT	• Social support (practical)<br>• Prompts/cues<br>• Remove aversive stimulus<br>• Restructuring the physical environment<br>• Restructuring the social environment
	Maintain recognition of PCP as part of the care team from diagnosis through to survivorship	Intuitive strategy	-		✓	CFIR, Engaging – Innovation deliverers	BCT	Social support (unspecified)
	Involve family/carers	Intuitive strategy	-		✓	CFIR, Engaging – Innovation recipients	BCT	Social support (practical/emotional)
	Utilise recall/reminder systems	Intuitive strategy	-		✓	CFIR, Engaging – Innovation recipients	BCT	Prompts/cues
	Provide health education and promote continuity of care	Intuitive strategy	-		✓	CFIR, Engaging – Innovation recipients	BCT	Information about health consequences
	Problem solve to support patient engagement and adherence	Barrier identified	Patients' financial capacity; Access to	✓		TDF, Environmental context and resources (individual)	BCT	Problem solving

Figure 3. Development		Evidence		Strategy development or alignment using theoretical framework				
				Recommendation development process				
Level	Recommendation	Evidence source	Related barrier/facilitator (if applicable)	*Evidence-informed*	*Thematic/ intuitive*	Determinant	Selected strategy taxonomy	Evidence-informed/aligned strategy
			services for rural and remote patients					
	Access to flexible PCP	Intuitive strategy	-		✓	CFIR, Engaging – Innovation recipients	BCT	Social support (practical)
	Reframe attitudes toward health care	Barrier identified	Young people avoidance	✓		TDF, Emotion	BCT	Framing/reframing
Process: Shared Care	Employ diverse telehealth modalities	Intuitive strategy	-		✓	CFIR, Adapting	ERIC	Change physical structure and equipment
	Propose more adequately funded Medicare billing items for survivorship care	Barrier identified	-	✓		CFIR, Policies and laws	ERIC	Make billing easier
	Provide adequate lead times for appointment planning	Intuitive strategy	-		✓	CFIR, Adapting	ERIC	Capture and share local knowledge
	Invite PCP to attend multi-disciplinary case review	Intuitive strategy	-		✓	CFIR, Adapting	ERIC	Change service sites; promote network weaving
	Promote transparent scheduling practices	Barrier identified	-	✓		CFIR, Structural characteristics - work infrastructure	ERIC	Capture and share local knowledge
	Conduct small scale tests of joint consultation approaches	Barrier identified	-	✓		CFIR, Structural characteristics - work infrastructure	CFIR, Structural characteristics - work infrastructure	Conduct cyclical small tests of chang
Survivorship care plan	Include health providers contact information		Suggestions, modifications or	✓	✓	CFIR, Design and Complexity	ERIC	Promote adaptability

Figure 3. Development		Evidence		Strategy development or alignment using theoretical framework				
				Recommendation development process				
Level	Recommendation	Evidence source	Related barrier/facilitator (if applicable)	*Evidence-informed*	*Thematic/ intuitive*	Determinant	Selected strategy taxonomy	Evidence-informed/aligned strategy
	Use visual formatting techniques (e.g., tables, numbered/dot points etc)	'Think aloud' activity	adaptations to content, format and structure of the survivorship care plan					
	Provide rationale for health recommendations							
	Keep care plan concise and avoid lengthy or dense paragraphs							
	Include information about patients' independence and functionality							
	Recognise PCP as part of the care team							
	Align care plan to support primary care workflow							
	Clearly delineate roles and responsibilities							
	Reinforce ongoing availability of clinic support							
	Include timing, frequency, priority and associated cost of recommendations							
	Include medication contraindications, cessation reasons and dates							
	Include information about patients' social history and status							
	Include family/carer contact information							
	Include clinically relevant and contextual details to support interpretation and care coordination							

Figure 3. Development		Evidence		Strategy development or alignment using theoretical framework				
				Recommendation development process				
**Level**	**Recommendation**	**Evidence source**	**Related barrier/facilitator (if applicable)**	*Evidence-informed*	*Thematic/ intuitive*	**Determinant**	**Selected strategy taxonomy**	**Evidence-informed/aligned strategy**
	Define healthcare services not universally known							

Acronyms: Consolidated Framework for Implementation Research; TDF, Theoretical Domains Framework; ERIC, Expert Recommendations for Implementing Change; BCT, Behaviour Change Technique; GP/PCP, General Practitioner/Primary Care Physician; CPD, Continuing Professional Development; SCP, Survivorship Care Plan.
