# Supplementary material for: Implementing childhood cancer survivorship care plans: qualitative perspectives and experiences from Australian primary care physicians
Source: Fam Med Community Health. 2026 Nov 5;14(Suppl 1):e004002. doi: 10.1136/fmch-2026-004002 (PMC13756439; doi:10.1136/fmch-2026-004002)
Supplement: Supplementary file 2 [file fmch-14-Suppl_1-s005.pdf]

Example revised ‘Engage’ survivorship care plan

The revised survivorship care plan presented in this figure is intended as an illustrative example of potential adaptations informed by PCP feedback and is not validated. It should not be used without further co-design, review, and endorsement by relevant stakeholders.

1. EXECUTIVE SUMMARY

Letter head: hospital branding, oncologist and survivorship nurse name, and contact information

Demographics: patient name, medical record number, next of kin, date of birth, contact information, risk stratification (risk of late effects)*, and blood group

Diagnosis: cancer type, grade, site, date of and age at diagnosis, protocol, date of treatment completion, relapse/second malignancy

Specialists: treating oncologist, and current adult/paediatric specialists names and contact information, parent/carer details, disability provider information; transition from paediatric to adult specialist service information including contact details

Request to Primary Care Physician: Thank the PCP for servicing the care plan, highlight need for multiple PCP appointments (where appropriate), numbered pointed requests by priority for primary care physician to action including timing, frequency, and whether ongoing specialist involvement is indicated (reference to supporting information in SCP in brackets, e.g., see page 6 systems review)

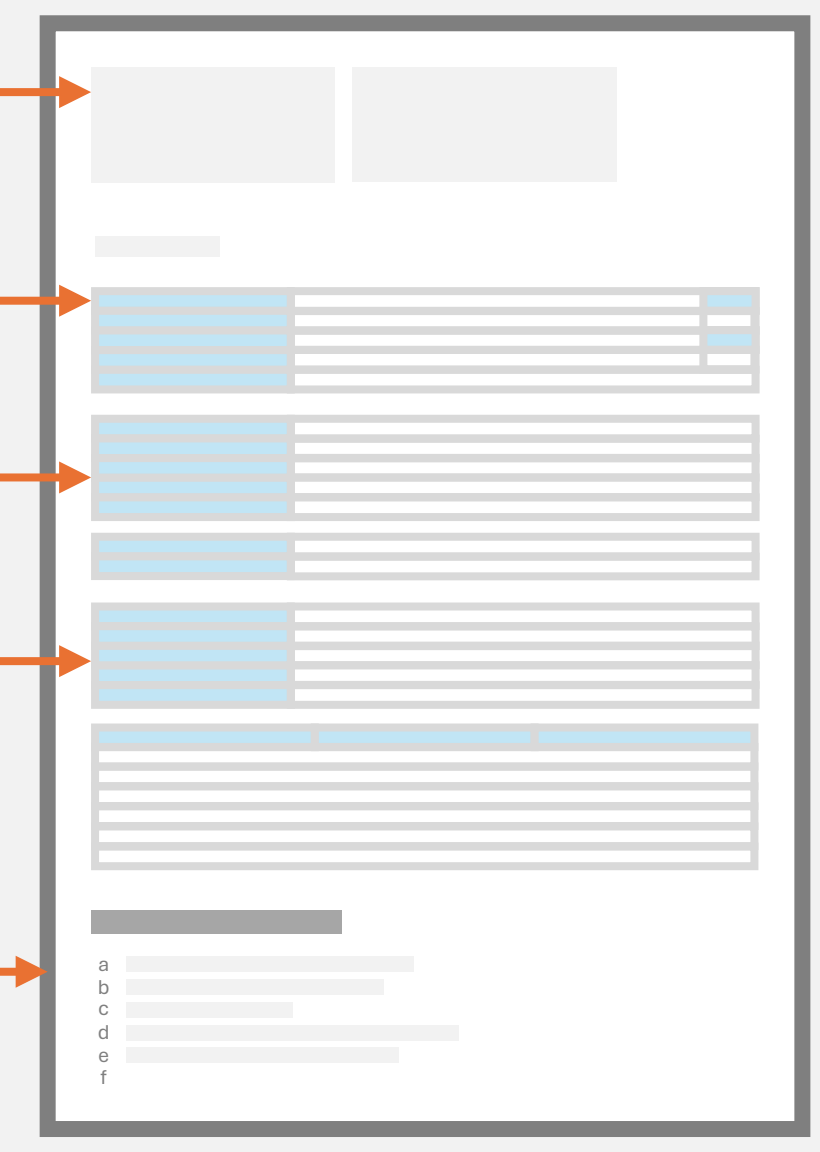

2. CANCER HISTORY

Cancer history: introduction to ‘Engage’ program, written summary of diagnosis, presentation, complications, treatment, post-treatment complications and last oncology review, patients’ current remission status, brief social history

Complications: numbered list of known late complications of cancer and its treatment (dates for each in brackets)

3. ASSESSMENT SUMMARY

Assessment and summary of recommendations: composition of multi-disciplinary team, written summary of multi-disciplinary team assessment and recommendations for care

Plan for follow-up in Survivorship Clinic: recommendations for ongoing Survivorship Clinic review

Patient education about survivorship: summary of patient education provided by survivorship nurse at the Survivorship Clinic

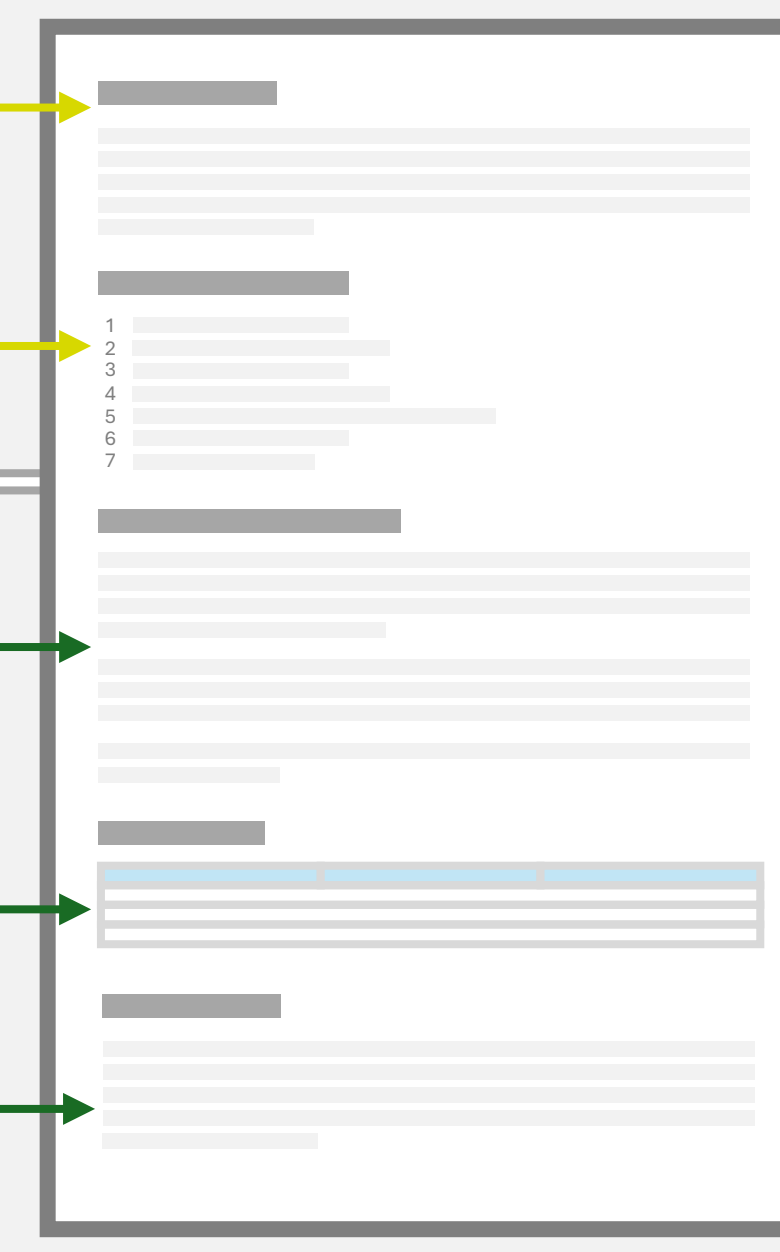

4. FOLLOW-UP/SCREENING

Follow-up visit/screening: visit/test (e.g., primary care, endocrine, social work), priority for follow-up, frequency (e.g., yearly), next due and any further notes

Health screening assessment: bowel screening, skin check frequency, echocardiogram, mammograms and any associated out of pocket costs

Psychosocial concerns

Health behaviours and lifestyle recommendations: diet, exercise, avoidance of alcohol, sun exposure, and vaping/smoking

Link to the Children’s Oncology Group (COG) guidelines

Explicit point of contact for PCP queries and reminder to contact clinic if condition worsens

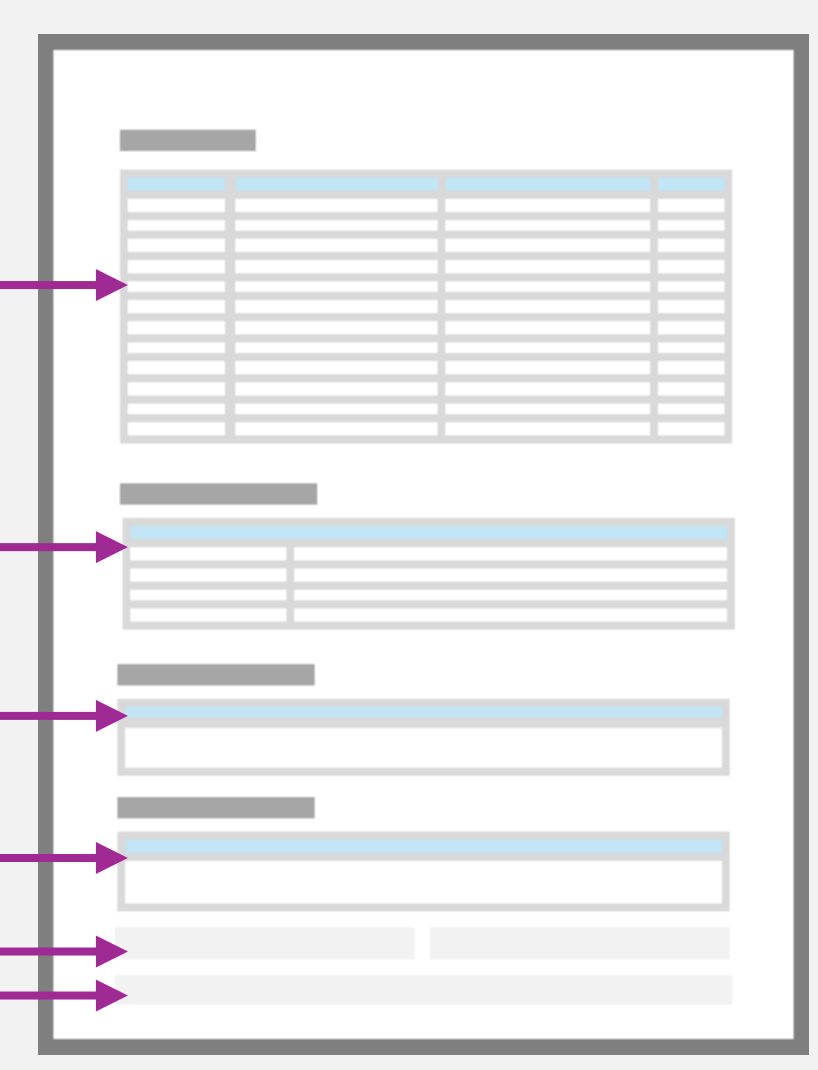

5. TREATMENT SUMMARY & BACKGROUND INFORMATION

Treatment summary: surgery (date, surgeon, procedure), chemotherapy (date, drug name, state whether high or low dose), radiotherapy (date, site, fractions, total dose, state whether high or low dose), administering hospital

Relevant other medical history including pre-existing conditions

Background information: brief summary of triage, assessment and follow-up process at the Survivorship Clinic

Current health status including level of functionality and independence

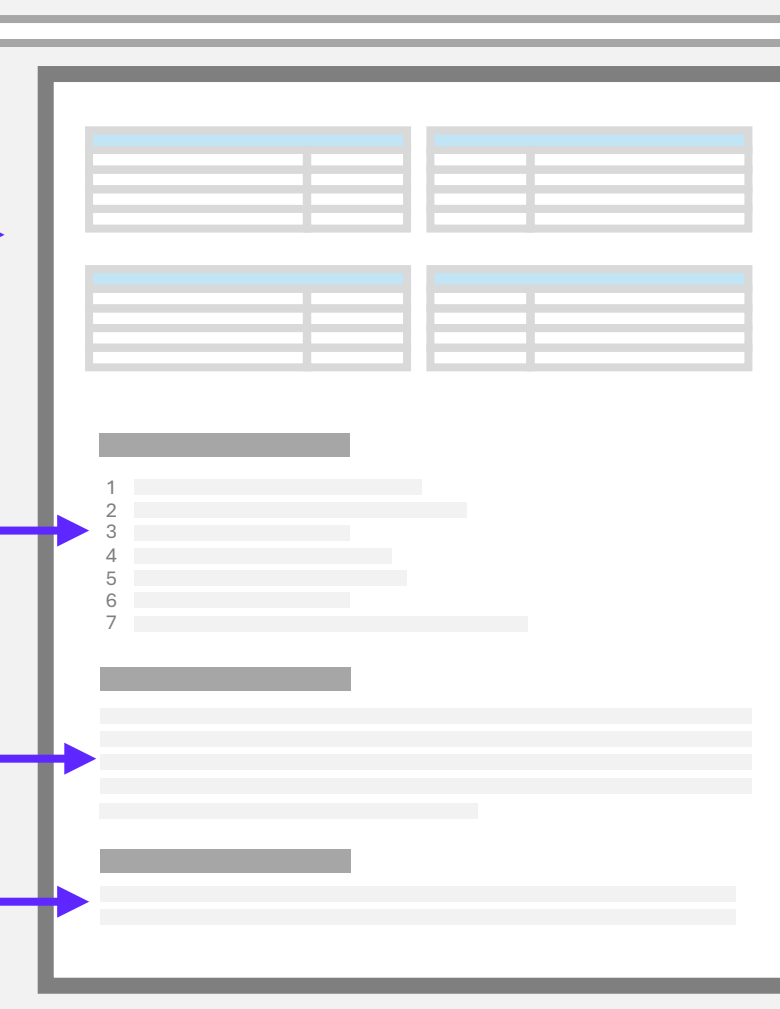

Systems review: e.g., cardiac, respiratory, audiology, eyes, endocrine, skin, neurology, gastrointestinal, musculoskeletal, reproductive health

Health behaviours: diet, dental, smoking/vaping/ recreational drugs, alcohol, sun protection, exercise/ leisure, health screening

Social history: relationship status, living arrangements, education summary, employment/occupation

Psychosocial review (flagging specific mental health concerns)

Family history of cancer

Medications, immunisations

Physical examination

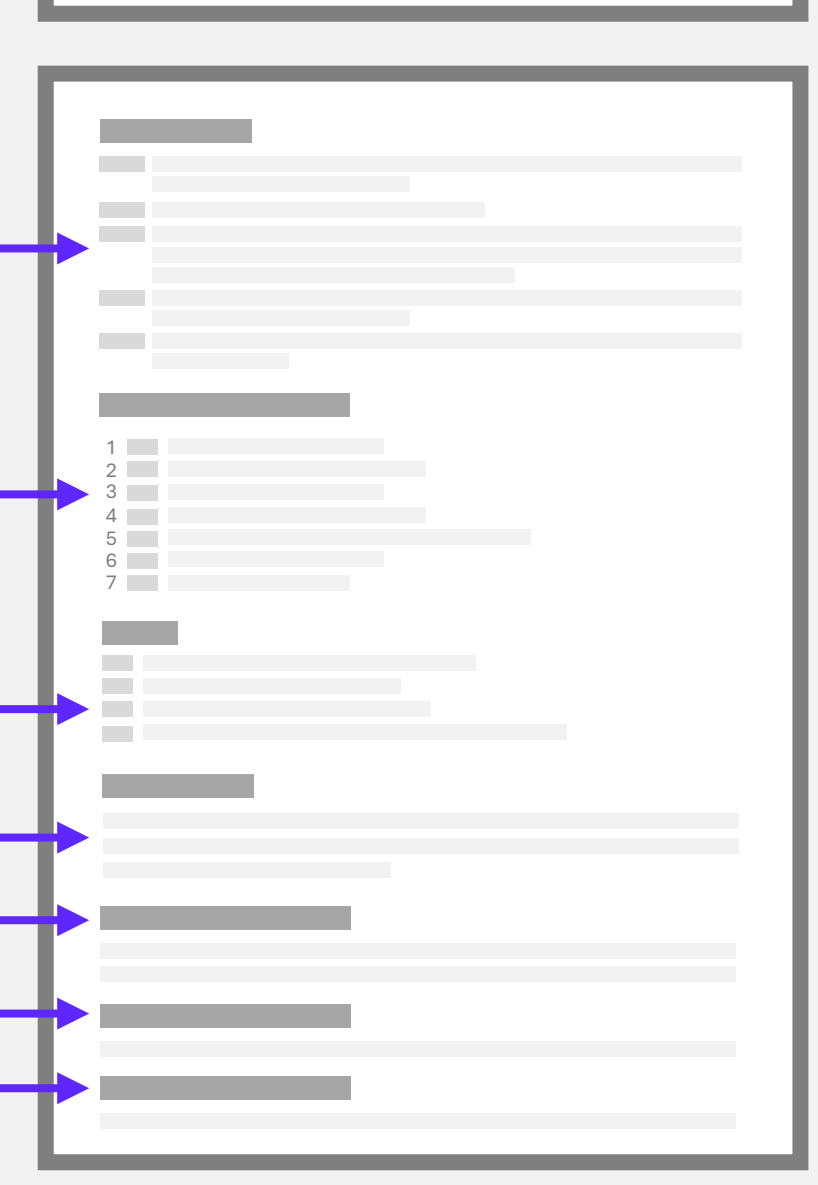

***Provide the following footnote within the care plan:** For guidance on interpreting treatment intensity and risk stratification, see Kazak, A.E., et al. (2012), A revision of the intensity of treatment rating scale: Classifying the intensity of pediatric cancer treatment. Pediatr. Blood Cancer, 59: 96-99. https://doi.org/10.1002/pbc.23320
