# Supplementary material for: Implementing childhood cancer survivorship care plans: qualitative perspectives and experiences from Australian primary care physicians
Source: Fam Med Community Health. 2026 Nov 5;14(Suppl 1):e004002. doi: 10.1136/fmch-2026-004002 (PMC13756439; doi:10.1136/fmch-2026-004002)
Supplement: Supplementary file 3 [file fmch-14-Suppl_1-s001.docx]

**Supplementary file 1: COREQ checklist**

The Consolidated Criteria for Reporting Qualitative Studies (COREQ): a 32-item checklist [1]

This study has been written in according with the COREQ checklist. Items not directly addressed in the main text of the manuscript are further explicated here.

| **No** | **Item** | **Description** | **Page No.**  **(N/A if not referenced in the manuscript)** |
| --- | --- | --- | --- |
| **Domain 1: Research team and reflexivity** | | | |
| *Personal Characteristics* | | | |
| 1. | Interviewer/facilitator | JE | Title page, Page 9 |
| 2. | Credentials | B. Psych (Honours) | Title page |
| 3. | Occupation | PhD Candidate | Page 9 |
| 4. | Gender | Male | N/A |
| 5. | Experience and training | Completed qualitative research coursework as part of his undergraduate degree. Has previously conducted qualitative research including focus groups in healthcare settings. Received guidance and mentorship from CM, JM, NT and CS who are all experienced qualitative researchers. | N/A |
| *Relationship with participants* | | | |
| 6. | Relationship established | The interviewer had no relationship to any participants before the interviews. PCPs who had referred their patient to participate in the ‘Engage’ intervention were initially approached by contacting their practice and emailing a study information sheet. All other PCPs were informed about the study through primary care networks, social media or snowballing techniques. Participants were informed prior to the interview that their participation was voluntary. Participants were remunerated for completing an interview and provided a certificate of completion evidencing their participation. | No relationship was established prior to interviews.  Further information is found in Fig 2, page 9. |
| 7. | Participant knowledge of the interviewer | Participants were informed that the interviewer was a PhD candidate who was interested in understanding their perspectives toward a survivorship care plan developed for patients who had participated in the ‘Engage’ survivorship intervention. | All participants were provided with an information sheet.  Page 9: *“All PCPs interviewed were emailed a copy of the participant information sheet…”* |
| 8. | Interviewer characteristics |  |  |
| **Domain 2: Study design** | | | |
| *Theoretical framework* | | | |
| 9. | Methodological orientation and Theory | We drew on implementation science determinant frameworks to guide data collection and analysis of barriers and facilitators. Specifically, CFIR 2.0 (incorporating COM-B and TDF) was selected for its suitability to our aims, enabling exploration of individual, organisational, and system-level determinants. Behaviour change taxonomies (ERIC, BCTs), were used to generate recommendations. Qualitative methods were underpinned by cognitive interviewing (via a ‘think aloud’ exercise’), and phenomenology to understand participants lived experiences. | Page 10 |
| *Participant selection* | | | |
| 10. | Sampling | Included purposive sampling via physicians referring patients to the ‘Engage’ program, snowballing through physicians that had participated in interviews, and convenience sampling. | Fig 2., page 9 |
| 11. | Method of approach | Participants were approached via telephone, fax and email. | Fig 2., page 9 |
| 12. | Sample size | Sample size was guided by principles of information power. We initially aimed to recruit 15 physicians. | Pages 9-10 |
| 13. | Non-participation | For purposive sampling procedures, 42 PCPs were approached from Engage, 37 did not respond to the invitation. Of the 5 PCPs that responded, 2 opted out (no reason provided). | Page 11 |
| *Setting* | | | |
| 14. | Setting of data collection | Australian primary care practices. Most were private (90%) and located in metropolitan areas (75%). | Study context described Page 9 and in Table 1 Participant characteristics |
| 15. | Presence of non-participants | All interviews were conducted by an implementation science PhD Candidate (JE). There were no other individuals present apart from the participant. | Page 9 |
| 16. | Description of sample | 14 (70%) PCPs were female, 13 (65%) completed their primary medical training in Australia and 11 (55%) practiced in the state of New South Wales with representation from all other Australian states and territories except one. PCPs reported an average of 8.5 (range, 1.5 – 20) years of experience. The median number of survivors that PCPs could recall treating within their practice over the prior 2-year period was between 1-2 (45%). | Page 5, 11 and Table 1 |
| *Data collection* | | | |
| 17. | Interview guide | The semi-structured interview guide was initially informed by the Consolidated Framework for Implementation Research and refined through consultation with a primary care physician (ETB) and pediatric oncologist (RC). The interview was piloted with two primary care physicians (ETB, RL) prior to recruitment. | Page 10, Supplementary file 2. |
| 18. | Repeat interviews | No repeat interviews were carried out. | N/A |
| 19. | Audio/visual recording | Interviews were conducted via teleconferencing (i.e., Zoom or Microsoft Teams) or telephone. | Page 9 |
| 20. | Field notes | JE wrote brief notes after completing each interview. | N/A |
| 21. | Duration | Interviews ranged from 38 to 59 minutes in duration. | Page 11 |
| 22. | Data saturation | Information power was reached after a total of 20 PCP interviews across both recruitment sources. | Page 11 |
| 23. | Transcripts returned | No transcripts were returned to participants for comments and/or corrections. | N/A |
| **Domain 3: Analysis and findings** | | | |
| *Data analysis* | | | |
| 24. | Number of data coders | Two independent coders (JE, SL) deductively coded ~20% of transcripts to the domains and constructs of the CFIR 2.0/TDF. Once a codebook was developed, the remaining coding was completed by JE. | Page 10 |
| 25. | Description of the coding tree | Directed content analysis was performed, so the coding tree was established using the domains and constructs of the CFIR2.0/TDF. Thematic codes are described in Table 2 providing themes and subthemes along with implementation determinants. | Page 10, Supplementary file 3 |
| 26. | Derivation of themes | A hybrid deductive-inductive approach was undertaken, meaning analysis was first informed through a deductive approach to an established implementation science framework, but also inductively through the generation of thematic labels derived from the data. | Page 10 |
| 27. | Software | Qualitative analysis was performed using NVivo 14. | Page 10 |
| 28. | Participant checking | Participants were not asked to provide feedback on the findings. | N/A |
| *Reporting* | | | |
| 29. | Quotations presented | Participant quotes are primarily presented in Table 2 and supplementary files 6 and 7, and each participant was identified by their sex, setting and years of experience. | Table 2, and Supplementary files 6 and 7. |
| 30. | Data and findings consistent | - | Table 2, and Supplementary files 6 and 7.  Pages 11-15 |
| 31. | Clarity of major themes | Implementation factors were organised into major and minor themes. All other findings were organised according to the four study aims. | Table 2, and Supplementary files 6 and 7.  Pages 11-25 |
| 32. | Clarity of minor themes | Implementation factors were organised into major and minor themes. All other findings were organised according to the four study aims. | Table 2, and Supplementary files 6 and 7.  Pages 11-15 |

Reference:

1.	Tong A, Sainsbury P, Craig J. Consolidated criteria for reporting qualitative research (COREQ): a 32-item checklist for interviews and focus groups. Int J Qual Health Care. 2007;19(6):349–57.
