# Supplementary material for: Implementing childhood cancer survivorship care plans: qualitative perspectives and experiences from Australian primary care physicians
Source: Fam Med Community Health. 2026 Nov 5;14(Suppl 1):e004002. doi: 10.1136/fmch-2026-004002 (PMC13756439; doi:10.1136/fmch-2026-004002)
Supplement: Supplementary file 4 [file fmch-14-Suppl_1-s002.docx]

**Supplementary file 2.** Semi-structured interview schedule

| **Components** | **Instructions to interviewee** | **Questions and probes (CFIR Domain/Construct)** |
| --- | --- | --- |
| **1. SCP content and format (CFIR Domain, Innovation)** | Explain to participants that the survivorship care plan will be displayed on screen and reviewed together in sections. Ask participants to read what’s displayed on the screen aloud or in their mind, and to verbalise any immediate thoughts or feedback they might have about the letter (e.g., the font is too small, the information is too dense or is irrelevant). | 1a. Once the participant has finished reading a particular section of the letter, ask the following concurrent probes if they have not been addressed.   - How clear or unclear was the information here communicated? If unclear, could you please describe why or suggest how it could be improved? **(CFIR, Innovation – Complexity)** - How practical/usable/relevant or unpractical/unusable/irrelevant was the information presented here for you to know in order to treat your patient? **(CFIR, Innovation - Design)** - Was the level of detail provided here sufficient? Do you feel that more or less detail would be beneficial? **(CFIR, Innovation – Design)** |
|  |  | 1b. Once the participant has read through the entire survivorship care plan, ask the following retrospective probes:   - How clear or unclear does the language used in the letter convey which recommendations should be prioritised? **(CFIR, Innovation – Complexity)** - For example, the letter may contain prescriptive language, like “as needed”, “possible involvement with” which may imply that some recommendations are more important than others - How have the communication resources provided in the letter impacted your level of knowledge of how to get back in touch with cancer services? **(CFIR, Capability; TDF, Knowledge)** - Having received this letter, how likely is it that you would contact the survivorship clinic for more information if your patient began developing new late effects or a new (non-cancer related) condition (e.g., pregnancy)? **(CFIR, Inner setting – Communications)** |
| **2. Factors influencing implementation (CFIR Domain, Inner/Outer Setting, Individuals)** | Explain to participants that we will now explore barriers within their PCP practice that might affect the implementation of the letter recommendations. | 2a. Can you walk me through what happens when you receive a letter like this for one of your patients?   - Will you read the letter before seeing the patient? Is it too long to read during the consult? Will you only read the front page (i.e., executive summary)? Will you mark off when each recommendation is completed? Will you set up reminders for you/your patients in your PCP software? Do you have a system in place so that someone, either yourself or someone else, would monitor these reminders and contact patients if they do not follow-through themself? Will you ask your patients to see the practice nurse for things like bloods, screening? Are there any particular patients or recommendations that are harder to act on and why? |
|  |  | 2b. Given the time and resources available to you in a long consultation, do you think it is feasible to implement the recommendations made in the letter? **(CFIR, Individuals – Opportunity; TDF, Environmental context and resources)** |
|  |  | 2c. Do you think that receiving detailed information and recommendations in this way would change your confidence to provide care to patients who have recovered from cancer as a child? **(CFIR, Individuals – Motivation; TDF, Beliefs about capabilities)** |
|  |  | 2d. Patient adherence to a healthy lifestyle and participation in regular surveillance is one of the main goals of the ‘Engage’. What barriers do you think may impede patient adherence, and what strategies could be employed by our team to improve patients’ engagement with you? **(CFIR, Inner setting – Culture recipient-centredness; Individuals – Motivation; TDF, Beliefs about consequences)** |
|  |  | 2e. Are there any additional supports or resources you would need to implement the recommendations from this letter? **(CFIR, Inner setting - available resources)** |
|  |  | 2f. After receiving this letter would you like to receive further support from a survivorship nurse specialist at the long-term follow-up clinic to action the recommendations? **(CFIR, Outer setting – partnerships and connections)**   - For example, the recommendation is to refer to a local psychologist, but you live in the country and there are no good psychologists available, perhaps the hospital knows of a cancer-related telehealth psychosocial service they could refer the survivor to instead. |
|  |  | 2g. Would you like the opportunity to speak to the nurse specialist about a complex area of care before you discuss with your patient? **(CFIR, Outer setting – partnerships and connections)**   - For example, you weren’t confident regarding some of the genetic information, and you’d like someone else to provide that discussion with your patient |
|  |  | 2h. How available or accessible are community and specialist resources in your area? **(CFIR, Outer setting – Local conditions; Individuals – Opportunity; TDF, Environmental context and resources)**   - If unsure, availability may mean the number of clinics available for referral, and accessibility may mean your patients ease of access whether that be cost, wait times, distance to travel, responsiveness etc |
|  |  | 2i. How often does your patient’s financial situation prevent them from accessing community or specialist resources? How do you overcome these barriers? **(CFIR, Inner setting – culture recipient-centredness)** |
| **3. Educational and informational supports (CFIR, Inner Setting, Access to Knowledge and Information)** | Explain to the participant that you would like to understand their perspective on their need and preferences for childhood cancer survivorship educational materials. Provide participants with the example of a series of short videos focused on a specific topic (e.g., what to do in an annual PCP check-up, common endocrine issues etc) that would be brief (i.e., 4 minutes long) and when taken together could form a repository of broad information. | 3a. In your opinion, how helpful would a repository of short educational materials be?   - For example, short videos, webinars, lectures, one-page summaries, checklists |
|  |  | 3b. What format would be most useful to you? |
|  |  | 3c. What topics or challenges would be most beneficial to cover in these materials? |
|  |  | 3d. How could we best let you know where to find these materials?   - For example, would you like to be emailed a link, would you like materials to be disseminated through the Royal Australian College of General Practitioners, through Primary Health Networks, or through the children’s hospitals |
| **4. Shared model of survivorship care (CFIR Domain, Process)** | Explain to participants that shortly after receiving the survivorship care plan, from ‘Engage’, patients participate in a nurse consultation which provides health education and troubleshoots barriers to accessing care. Explain that while this consult is currently being led by the nurse, that we are exploring the possibility of involving PCPs to facilitate a more supportive and integrative shared care process for them and their patients. Provide participants with a brief operationalised example of how this might work: their patient would book a long appointment via the usual method, during the appointment their patient would arrive with a QR code to join a teleconference (e.g., Zoom), the nurse would go through each multi-disciplinary team recommendation, providing in-depth and tailored information with the opportunity to ask questions and troubleshoot recommendations that may be more difficult to facilitate. | 4a. How does the proposed joint consultation approach compare with the current model of care (i.e., the nurse leads with consult with no involvement from the PCP) **(CFIR, Process – Reflecting and evaluating)** |
|  |  | 4b. Are there any kinds of changes or alterations that you think will need to be made to this joint-consultation approach so that it will work effectively in your setting? **(CFIR, Process – Assessing needs innovation deliverers, Adapting)** |
|  |  | 4c. Apart from this proposed joint consultation, is there a potentially more effective way to facilitate a smooth transition from specialist to primary care for patients from ‘Engage’? **(CFIR, Process – Assessing context)** |
| **Acronyms:** CFIR, Consolidated Framework for Implementation Research; TDF, Theoretical Domains Framework; PCP, Primary Care Physician. | | |
