# Supplementary material for: Implementing childhood cancer survivorship care plans: qualitative perspectives and experiences from Australian primary care physicians
Source: Fam Med Community Health. 2026 Nov 5;14(Suppl 1):e004002. doi: 10.1136/fmch-2026-004002 (PMC13756439; doi:10.1136/fmch-2026-004002)
Supplement: Supplementary file 5 [file fmch-14-Suppl_1-s006.docx]

**Supplementary file 6.** Primary care physicians’ preferences for survivorship care plan content and format

| **Information domain** | **Existing features:**  *Barrier (-), facilitator (+)* | **Suggested adaptations** | **Illustrative quote (participant demographics)** |
| --- | --- | --- | --- |
| **1. Treatment summary** | | | |
| *1a. Letter head* | - Access to hospital contact numbers (+) - Branding (+) - Care plan is dated (+) | - Remove provider number and oncologist qualifications to save space | - “I find it so hard to know who to contact when you have a question. So having the email and the phone numbers and the fax is really helpful” (female, metropolitan, 6 years’ experience) |
| *1b. Patient demographics* | - Patients’ blood type (-) - Risk stratification (-) - Patient demographics (+) - Next of kin (+) | - Qualify risk stratification | - “…the risk stratification for me probably didn't mean too much. I felt like that was probably more the cancers service of knowing what that meant, so that maybe needs clarification” (female, metropolitan, 6 years’ experience) |
| *1c. Diagnosis* | - Primary cancer site, diagnosis and date of diagnosis (+) - Age at diagnosis (+) - Treatment date completed (+) - Protocol (-) | *No adaptations* | - “I also like that you have the age of diagnosis on the right-hand side, so it stands out, instead of just scrolling and scrolling, trying to find out” (male, metropolitan, 6 years’ experience) |
| *1d. Relapse/ second malignancy* | - Relapses/second malignancy (+) | *No adaptations* | - “I thought it was terrific that it clearly highlights if there's been any, you know, relapses or malignancies” (female, metropolitan, 6 years’ experience |
| *1e. Specialists* | - Specialists’ names are provided (+) - Health services that are not universally known (e.g., Rehab2Kids) (-) | - Distinguish between adult and paediatric specialists - Include contact information for specialists - Include details for National Disability Insurance Scheme provider - Include details for parent/carer | - “… if we receive a casual letter from a specialist, it's going to be, oh, hi, I'm the paediatrician. I saw this child. Okay, please refer to neuro or whatever. They won't have all the specialist numbers there, so I will be asking, who's your neurologist? …we end up spending time on trying to find who's the neurologist, etc. So, tables and the numbers the names of all the specialists. That's a very good idea” (male, metropolitan, 6 years’ experience) |
| *1f. Surgery details* | - Cancer treatment protocol (+/-) - Position of treatment protocol (-) - Surgery dates (+) | - Add treatment dates and chemotherapy cycle count - Qualify treatment dosages - Include administering hospital for treatment - Re-position treatment protocol to the end of the care plan | - “I don't think I'd be sitting next to the patient and the patient's family saying, Okay, I know you've had cisplatin and cyclophosphamide and vincristine, and you've had 54 rays or whatever I don't even know what GY stands for...so for me, do I necessarily need to know you have this many fractions, this dose of radio(therapy)” (female, metropolitan, 4 years’ experience) |
| *1g. Chemotherapy details* |  |  |  |
| *1h. Radiotherapy details* |  |  |  |
| **2. History** | | | |
| 2a. Cancer history | - Introduction to Engage intervention (+) - Cancer history (+/-) | - Clarify patients’ current health status - Include a brief social history - Expand cancer history with relevant details (e.g., number of shunts, cause of anaphylaxis, baseline for cognitive deficits etc) | - “history - that is very useful. It says, it mentions what happened, what symptoms, what we did, what went away. I'm assuming remission” (male, metropolitan, 15 years’ experience) - “The cancer history is good, probably a little bit, maybe bit too much information, as what happened in the hospital, there are specifics of the procedure is probably a little bit less relevant” (male, metropolitan, 8 years’ experience) - “With the cancer history…it seemed like everything happened in 2015… he remained stable with XYZ and, we didn't need to see him more regularly and we’re happy to just see him yearly because he's stable, and we're not concerned. Just because at the end, it's kind of like all these things happened …. then it's just abruptly like, oh, he was last seen, four years ago… and that might be because he's an adult now. But sort of maybe clarifying what the status is now” (female, metropolitan, 3 years’ experience) |
| 2b. Known late complications of cancer and its treatment | - Known late complications of cancer and its treatment (+) | - Include dates of complication from cancer and treatment in brackets | - “I think it was really helpful to know the complications as well and what needs to be done in terms of optimizing people's health post those complications…because a lot of the time when they've had such a prolonged admission, they've undergone several procedures, it's really hard to understand the timeline of it. So it's good to actually have a bit more detailed information as to what he went through and the complications that happened, that we can be aware if there's anything that we need to follow up one based on the complications that occurred and procedures” (female, metropolitan, 8 years’ experience) |
| **3. Assessment** | | | |
| 3a. Assessment and summary of recommendations | - Summary of recommendations (+) - Clinicians involved in MDT (+) - Family care of shunt (+) - Reminder to encourage positive lifestyle and social behaviours (-) | - Specify provider responsible for shunt management | - “…the importance of the patient in the family knowing about the care of the shunt. That probably should be put in its own paragraph, because I think that's quite, I think everything is important” (female, metropolitan, 4 years’ experience) |
| 3b. Plan for follow-up in the survivorship clinic | - Plan for follow-up in the survivorship clinic (+) - Patient’s transition plan from paediatric to adult specialist services (+) | - Consolidate information about specialists involved in patient care into one table - Define healthcare services that are not universally known - Provide specialist contact information | - “… [who’s] going from paediatrics to adults and who the recommendation would be that is super useful, especially … for younger GPs who move around more often, if you don't know who the people are in a certain area, it's like, oh, who should we refer [for a] cancer recovered child too. And you're like, I'm going to pick a name at random. So it's super helpful for them to say this is who we would recommend specifically for someone who's got the cancer history in terms of you know endocrine or neurology or whatever” (female, metropolitan, 3 years’ experience) |
| **4. PCP requests** | | | |
| Requests to PCP | - Priority of PCP requests (-/+) - Ongoing specialist referral (-) - Position of PCP requests (-) - Rationale behind recommendations (i.e. reason for why request is needed) (-) - Actionability of recommendation (i.e. understand what needs to be actioned) (+) | - Detail method of screening in PCP requests - Highlight need for multiple PCP appointments - Include timing and frequency for PCP requests - Indicate ongoing specialist involvement (where appropriate) - Number PCP requests by priority - Reference PCP requests to supporting information in SCP (e.g., in brackets, see *systems review*) - Position PCP requests at the start or end of the care plan - Thank the PCP for servicing the care plan - Avoiding splitting requests across pages - Clarify what should be done in a yearly physical examination | - “I wasn't sure which one's most important. In terms of priority… blood pressure, growth, things like that. I think I might assign … the one that's more important than the other would be really good because, as a GP, often we're like, let's deal with the most important agenda first. Because often we got 15 minutes…” (female, metropolitan, 2 years’ experience) - “…with the cardiac echo every 5 years… what am I exactly looking for on the echo? I can order it, but at the same time I don't really understand the reason for doing the monitoring is there anything I should be monitoring for specifically on the echo? … I think it links somewhere where there was the survivorship guidelines. I clicked on that, and that was like a 300 page document, and in it I did find this is what the echo is for…but at the same time [the SCP] may not necessarily be that helpful in terms of actually the why of why I'm doing all of this…” (female, metropolitan, 3 years’ experience) - “…so just dot points, things we need to check off, all the things that we need to do and the patient needs to follow up on, laid out, really succinct in easy to see formats” (male, metropolitan, 8 years’ experience) |
| **5. Background** | | | |
| 5a. Patient education about survivorship | - Patient education about survivorship (-) - Health behaviours (+) - Immunisations (+/-) - Medications (+) - Psychosocial review (+) - Psychosocial scores (-) - Relevant other medical history (-) - Reproductive health (+) - Social history (+) - Systems review (+) | - Background health information and review should be separate from SCP - Clarify patients’ level of functionality - Include contraindications for medications - Flag specific mental health concerns - Include pre-existing conditions in relevant other medical history and refer to late complications above - Provide dates medication ceased - Provide reasons for why medications have ceased - Qualify psychosocial scoring | - “So the background information systems review, health behaviours, psychosocial review, to be honest, most likely would skim so again, I think if there's a few salient points that's important to highlight them, or to have a just an executive summary, half a page, one page executive summary, and then have the rest of information as an addendum or attached, I think would be probably helpful” (male, metropolitan, 8 years’ experience) - “they'd said ceased Metformin, … I'm getting the picture that he's potentially rebelling and doesn't want to take his Metformin and wants to drink alcohol and is fed up with health care altogether … But maybe if there's something where they've self-ceased…just being like, because couldn't afford or whatever” (female, metropolitan, 3 years’ experience) - “we're meant to update like social history and things like that and constantly check in about smoking and alcohol and look, let's be honest, we are often busy and we often do forget… so having that can be a helpful check in for me” (female, metropolitan, 6 years’ experience) - “you make my life much easier by adding the social history. Because this is really important for us as GPs, so it's much higher priority me to read what's happening with the family. Who they who they are receiving support? Who do they provide support as well? Rather than, do they have 15 grams of this medication 15 years ago” (male, metropolitan, 6 years’ experience) |
| 5b. Relevant other medical history |  |  |  |
| 5c. Background information |  |  |  |
| 5d. Current health status |  |  |  |
| 5e. Systems review |  |  |  |
| 5f. Health behaviours |  |  |  |
| 5g. Social history |  |  |  |
| 5h. Psychosocial review |  |  |  |
| 5i. Family history of cancer |  |  |  |
| 5j. Medications |  |  |  |
| 5k. Immunisations |  |  |  |
| 5l. Reproductive health |  |  |  |
| 5m. Physical examination |  |  |  |
| **6. Follow-up/screening** | | | |
| 6a. Follow-up schedule | - Specificity of follow-up instructions (-) - Goals of specialists involved (+) - Supports inputting recall/reminder systems (+) | - Identify priority specialists for follow-up - Include frequency of ongoing specialist assessment - Include PCP role in follow-up table - Include reminder to contact long term follow-up care if condition worsens | - “…it’s oh good information let me add all the reminders through this one…” (female, metropolitan, 14 years’ experience) - “I can see straight away this is endocrinologist, this is what they're doing, this is their follow up. It's very easy to see. So that table format structure really good, and how often they need follow up. Makes it easy, because then we can, when we put the reminder, then we put the recall reminders for this time period, rather than paragraphs. That table is a very practical way and very easy to follow” (female, metropolitan, 2 years’ experience) - “…what if it's more precise? Because as per endocrinologist, as per ophthalmology, it means pretty much nothing to me” (male, metropolitan, 16 years’ experience) - “When I see the word comprehensive history and physical examination by GP, I would just target, do a targeted examination. So if it's a brain and cranial nerves, and then health relate-wise, heart and chest, like cardio respiratory, but often it's targeted examination, so it'd be good if they could say about which ones are important for us to do” (female, metropolitan, 2 years’ experience) |
| 6b. Health screening assessment | - Screening guidelines and links to information (+/-) | - Clarify out-of-pocket costs for non-routine screening - Include screening schedule in follow-up table | - “I would wonder about the cancer screening recommendations as well. Would that be covered by bowel cancer screening from age 30? If I ordered a blood test for that would that be covered by Medicare, or would they be paying out of pocket? Because normally for screening that's only covered now from 45 years of age” (female, metropolitan, 6 years’ experience) |
| 6c. Psychosocial concerns | - Psychosocial normalisation (+) | *No adaptations* | - “Well, I quite like the psychosocial stuff, I like that box. Just saying it's normal to feel all those challenges” (female, metropolitan, 4 years’ experience) |
| **7. Overarching/general** | | | |
| Overarching/ general feedback or suggestions | - Abbreviating terms that are not universally known (-) - Repeating information across multiple sections (-) - Dense or lengthy content (-) - Uniform colours (-) | - Apply visual formatting techniques (e.g., dot points, underlining, tables) for readability throughout - Clarify point of contact for PCP queries - Highlight urgent information at the end - Include functional and independence status - Structure SCP to mirror PCP consult format: history, examinations, investigations | - “I think that the most important things are that the letters are as brief as is reasonable, that they're clearly structured” (female, rural, 11 years’ experience) - “…the paragraphs are a little bit intimidating” (male, metropolitan, 8 years’ experience) - “So I think tabulated format is very helpful in trying to kind of, I guess, read information quickly, and absorb information quickly” (female, metropolitan, 8 years’ experience) - “I just want some functional status. So is there other learning challenges or their mobility? Are there barriers to studying working? … And how’s it changing? So if you guys have followed them for three years already, like 12 years, have seen things progress like that trajectory is really useful for me…what's happened to the parents?” (female, metropolitan, 15 years’ experience) |
| Deductive coding to the Consolidated Framework of Implementation Research (CFIR 2.0) across all SCP components mostly mapped to the *Innovation* *Design* and in some instances *Innovation Complexity* constructs [24].  **Acronyms:** PCP, Primary Care Physician; SCP, Survivorship Care Plan; MDT, Multi-Disciplinary Team. | | | |
