# Supplementary material for: Implementing childhood cancer survivorship care plans: qualitative perspectives and experiences from Australian primary care physicians
Source: Fam Med Community Health. 2026 Nov 5;14(Suppl 1):e004002. doi: 10.1136/fmch-2026-004002 (PMC13756439; doi:10.1136/fmch-2026-004002)
Supplement: Supplementary file 6 [file fmch-14-Suppl_1-s007.docx]

**Supplementary file 7.** Themes, CFIR 2.0/TDF constructs, and illustrative quotes related to survivorship care plan implementation

| **Subtheme:**  *Barrier (-), facilitator (+)* | **Framework, construct** | **Illustrative quote (participant demographics)** |
| --- | --- | --- |
| **Theme 1: Structural and systems level factors (outer setting)** | | |
| Access to community care and specialist resources (-) | *CFIR, Local conditions* | 1a) “I've got patients who will die of bowel cancer before they get the scope because there's a two to three year waiting list, even if they have bowel cancer symptoms and positive for blood tests…there's no psychology access at all… it's so frustrating trying to get people to see specialists they need to see…” (female, metropolitan, 3 years’ experience) 1b) “Finding low cost or bulk billed services is much harder, much less available. Finding psychiatrists is very difficult.” (female, metropolitan, 15 years’ experience) 1c) “Access to specialists regionally is difficult. We do have a neurologist, who’s not taking new patients…we don’t have [an] ophthalmology service” (female, rural, 1.5 years’ experience) 1d) “So, psychiatrists and psychologists, it is impossible - there is such an access block” (female, rural, 11 years’ experience) 1e) “…for my country town, it's very limited, because we don't really have paediatric output system in town” (female, regional, 10 years’ experience) |
| Communications with hospitals/ specialists (-) | *CFIR, Partnerships & connections* | 1f) “The rejection of the referrals is a massive phenomenon in my area, because I understand [hospitals are] struggling. They don't have the capacity, [and] letters are being missed. We're using faxing, like we're in 1940s or 50s, instead of streamlining the transfer of information…I'll wait two or three weeks to receive the letter…. So it takes a number of weeks for the admin for the letters to be processed, not from my side, but from the hospital…I think the whole communication between primary care and secondary care should improve” (male, metropolitan, 6 years’ experience) 1g) “If the GP could receive the letter head ahead of time, and so they can be like, hey, can you book in the patient for a long appointment so that we can get started on discussing the letter and planning out what we need to do. But if the patient just kind of turns up with a letter, and they expect, all of these things, to be done, then they probably would walk away feeling pretty disappointed … part of it is also that communication and setting those patient expectations of this is going to be a long-term thing, because it also mentions on here seeing your GP at least once yearly. I worry that it makes the patient think this is just a once a year, 15-minute appointment [and] that everything's going to be done in that one appointment” (female, metropolitan, 3 years’ experience) |
| Patient advocacy and leveraging relationships with clinicians (+) | *CFIR, Partnerships & connections* | 1h) “I've sort of made connections with colleagues on the way, and so if something needs to happen urgently, I'll just send them a text and say, hey, can you help me sort this patient out? Depending on what it is, and usually that will happen” (male, metropolitan, 8 years’ experience) 1i) “The other thing is I will sometimes just call the registrar on call directly and be like, this is a situation they do not have any money, but they need this done. And if the reg is like no, just refer them they’ll wait, then I typically call back the next day and try to speak to a different reg and see if someone else is on call. And sometimes that helps. And they you kind of pull the right heartstrings and get them in, which is [explicative] medicine” (female, metropolitan, 3 years’ experience) |
| Access to survivorship nurse (+) | *CFIR, Partnerships & connections* | 1j) “…a lot of the shared care programmes that I participate in locally for obstetrics, for example, we've got a midwifery/GP liaison who will take our calls at set times through the week. And if I've got something that's not super urgent, but needs attention, and I'm not totally sure of how to navigate through… [if] it’s falling outside of some particular guideline, it's really nice to be able to bring somebody who knows the system publicly and who can help you navigate that so you don't feel so alone, working in general practice at times can be quite isolating for practitioners. So, to have a broader health team, with somebody who has a wealth of experience in that particular area, to say can I just pick your brains about this, I've got this person that were known to, you've done this lovely letter. This is their problem. Now, I think it's this, Or am I missing the mark here, and I need to be looking at something else? And then to have some suggestions on a pathway forward, if there's not an obvious local referral option, it is really valuable” (female, rural, 1.5 years’ experience) 1k) “…if the CNC or the CNS, or whoever is the point of call, if [I say] this has happened, and I'd like them to see the survivorship clinic again, and typically it's always the CNCs who are just like guns and so on the ball and super awesome in getting back to you…just having a number or an email address is super handy” (female, metropolitan, 3 years’ experience) |
| Ongoing eligibility for PCP management plans (+) | *CFIR, Policies & laws* | 1l) “…patients that can access our clinic, we can help them to fund the care through a chronic disease plan. It gives us regular reviews, which lets us chip away at these things. Some things won't have to be done right on the first day that they come in. This is a kind of annual plan, and some things might be even biannual. So, the clinic's accessible, the appointments are accessible, the costs are manageable” (female, metropolitan, 15 years’ experience) |
| **Theme 2: Practice-level factors (inner setting)** | | |
| Practice software constraints (-) | *CFIR, Information-technology infrastructure* | 2a) “…the problem with our practice software, you can't actually put in a recurring reminder, which would be very useful…when something like [this health recommendation] comes, we can put in a reminder for the next one. So say, in a year's time, needs a skin check, needs a full medical, but then once that comes around and that reminder is actioned, then we have to put in another reminder for the year after” (male, metropolitan, 13 years’ experience) |
| Centralised health record (+) | *CFIR, Information- technology infrastructure* | 2b) “[We] encourage young person’s to be registered with MyHealth record. So in that case every health professional can access, and you upload it into MyHealth record and with young person’s permission, we all can access the detailed information. So that's very important, especially young person can be very mobile, not necessarily stay in one location for very long. If they move to a different location, they will require a new GP” (female, metropolitan, 15 years’ experience) |
| **Theme 3: Provider-level factors (individuals, roles: innovation deliverers)** | | |
| Time constraints and pressures (-) | *CFIR, Opportunity, TDF, Environmental context and resources* | 3a) “I think the time pressure is just getting harder and harder to be able to do all of that stuff. It's, I think, the biggest thing that it comes down to is in general practice, you're taking a financial hit so it’s that balance that's really hard” (female, metropolitan, 6 years’ experience) 3b) “I guess the way that my practice is done, which is bulk billing, means that you only have limited time to do limited things. And I guess my concern would be that, and this is speaking very frankly and honestly, a yearly thyroid palpitation, I don't think that will ever be done, and because that's not something that I would do unless (a), the patient prompts me, or (b) it’s something that is part of something that I normally do anyway” (male, metropolitan, 8 years’ experience) |
| Knowledge of survivorship care (-) | *CFIR, Capability, TDF. Knowledge* | 3c) “I think the main issues we have right now is we don't know how far we have to do. And sometimes number one, I'm not a paediatric oncologist. So, there is my limitation in the knowledge having all those things that I can do to help, but at the same time, I wish I can help them to at least arrange some of the tests or get the pressure off from the hospital as well. And if we have these letters, so that I think everybody working with the family, with the survivor, we on the same trend, then we know where to go rather than making a lot of confusion to the patients and to their family” (female, rural, 2 years’ experience) 3d) “Yeah, it's a very foreign term especially GPs who are roughly around the same stage that I am at. I don't think a lot of us have ever heard the word survivorship, or cancer survivorship before” (female, metropolitan, 8 years’ experience) |
| Improved confidence through care plans (+) | *CFIR, Motivation, TDF, Beliefs about capabilities* | 3e) “…it's very helpful to have a list of the requirements, the appointments, follow up screening, and you know which ones we are to organise and which ones we need to refer to other specialties to provide. So that would definitely increase my confidence in terms of managing these patients appropriately” (Male, metropolitan, 8 years’ experience) 3f) “I think definitely would improve the confidence in that, at least, I know, look, this is kind of a guide as to what I can work off, and if have questions, then at least, I know, like, Hey, this is the specific question that I have in terms rather than like, I don't actually know what I'm doing [and] how I should I go about monitoring things” (female, metropolitan, 3 years’ experience) |
| Improved care coordination through care plans (+) | *CFIR, Partnerships and Connections* | 3g) “At the moment and how it has been for a long time, there is this big segregation between public hospital and general practices. They say that we're together, but we're not really together because often there is this lapse of information and we're often not included in the treatment plan. We often don't know what is happening when they review patients in their consultation rooms ... So having such a detailed letter is the dream because then I feel like I am part of that team. I feel like I respected and having an active role in this patient's life as opposed to being oh we’ll just use the GP to do whatever when you know we can't do it between our appointments” (female, metropolitan, 6 years’ experience) |
| **Theme 4: Patient-level factors (individuals, roles: innovation recipients)** | | |
| Financial capacity (-) | *CFIR, Opportunity, TDF, Environmental context and resources* | 4a) “We do see quite a few struggling parents that may have to save up and wait for pay to come in before they can actually see a specialist. And we've had patients having to reschedule cancer appointments because they just haven't got the money to see the specialist on that day.” (male, metropolitan, 8 years’ experience) |
| Access to services for rural and remote patients (-) | *CFIR, Opportunity, TDF, Environmental context and resources* | 4b) “…most of the time they need to get like a surgery, stuff like that, or even some of the rehab services is not available here. So, travelling to [Metropolitan Hospital 1] is two hours away from us. [Metropolitan Hospital 2] is at least four hours from us. And the close hospital that will be by plane. So, this is something that we are now facing and struggling with.” (female, rural, 2 years experience) |
| Young people avoidance (-) | *CFIR, Motivation, TDF. Emotion* | 4c) “…adolescents are notoriously bad at engaging with healthcare because they just want to be kids and enjoy their lives” (female, rural, 11 years’ experience) 4d) “I think that the person that has been to the hospitals receiving therapy as a young person like chemotherapy, radiotherapy might have affected their perspective of seeing the doctor, meaning I go to the doctor and something catastrophic will happen. I'll have to go to the hospital. I have to need an operation…So I think this will be a good bridging between like, Okay, this is your history, this is what the others did, and I'm totally aware, and we'll move forward together. So it might help with the relationship with GP and doctors in general.” (male, metropolitan, 6 years experience) 4e) “…They just get tired of being different. They get tired regular blood test appointments and all of these things. So they go on holiday…when they go to holiday, [I] can't they say, Oh, don't drink alcohol. So, it's a bit difficult to ask them, maintain the caring of your health, with the blood test, with medication, all of these things.” (female, 14 years’ experience) 4f) “I think one of the main challenges I foresee is that, children grow up, they move houses, they go to different schools, they may move to other cities or states, and so at some point, we're probably not likely to keep them as a patient forever, and when they change practices, then there's no real formal handover process, and we can request the files and records, but I can foresee that things might very easily get lost along the way, and the new GP they see may not have the these paperwork and may not know them as well, may not know where to look in terms of finding out this information…as they grow up… [they] think that's all behind us, and forget about the preventative measures, and follow up plans, and they may be less inclined to engage with healthcare…So that's from just a patient initiated screening perspective, sometimes that can be hard to approach. I mean, we can send out as many reminders and recalls as we know we can, but obviously, if they choose to ignore it, that's difficult to know to motivate them to come back.” (male, metropolitan, 8 years’ experience) |
| Family/carer support (+) | *CFIR, Opportunity, TDF, Social influence* | 4g) “A good support network at home. So family or close friends…Just to pick you up when you are down, go for a walk, keep doing everything, you're doing encouragement.” (female, metropolitan, 4 years’ experience) |
| Strategies to support engagement/ adherence | *CFIR, Engaging, Innovation recipients* | 4h) “I do think having a good GP really is important. And a GP that is flexible, like someone that's okay, well you’ve got 15 minutes, but I can take a half an hour like with you. So, someone that if you can check in regularly and you have a good rapport with the GP can really encourage the lifestyle changes” (female, metropolitan, 4 years’ experience) 4i) “We do put in recalls, using the practice software recall systems to remind people when they're due for appointments” (female, rural, 11 years’ experience) 4j) “Explaining maybe, what's the risk of this recurring, how long they need to be monitored for…are they at lifelong risk for having these things and needing five yearly cardiac echoes?...understanding the importance of that, because explaining that to the GP and explaining it to them is also important, because once they’re cancer free for a while, they feel like that's it, they're done.” (male, metropolitan, 8 years’ experience) 4k) “A lot of patients have their specialists that they've seen for a long time and probably view them as the experts in their care, and probably don't have a great understanding of the role of the GP and the importance of that continuity in terms of the preventative [health]. I think it's a big shift when you go from acute management and then transition to adulthood, and I think that role and the importance of the continuity sometimes they'll have had quite a few different GPs and go from one medical centre to another based on their availability. So I think that would be a big thing, really emphasizing the role as the GP in the continuity of care for them that they're coordinating the other specialists” (female, metropolitan, 6 years’ experience) 4l) “So theoretically, when they come for medications, we've been trained… [to say] oh, by the way, do you know you're due for this? So, yes, you’re right, it can be done opportunistically. So, when they come for medication, so if you attach it to something that they’re due for then it's much easier to get them to come, get them returning…we do have a recall system. So, the nurses do get involved in the recall system. They're pretty good with trying to follow up with patients to come back, but I think often you need a good incentive for them to come back like this is either really important for this reason, and if there's a financial barrier, then financial support. Not an issue to have a healthcare card, of course” (female, metropolitan, 2 years’ experience) |
| **Acronyms:** Consolidated Framework for Implementation Research; TDF, Theoretical Domains Framework; GP, General Practitioner/Primary Care Physician; CNC/CNS, Clinical Nurse Consultant/Clinical Nurse Specialist. | | |
